# Expanding the Ligand Classes Used for Mn(II) Complexation: Oxa-aza Macrocycles Make the Difference

**DOI:** 10.3390/molecules26061524

**Published:** 2021-03-10

**Authors:** Ferenc K. Kálmán, Viktória Nagy, Rocío Uzal-Varela, Paulo Pérez-Lourido, David Esteban-Gómez, Zoltán Garda, Kristof Pota, Roland Mezei, Agnès Pallier, Éva Tóth, Carlos Platas-Iglesias, Gyula Tircsó

**Affiliations:** 1 Department of Physical Chemistry, Faculty of Science and Technology, University of Debrecen, Egyetem tér 1, H-4010 Debrecen, Hungary; kalman.ferenc@science.unideb.hu (F.K.K.); nagywiki@gmail.com (V.N.); garda.zoltan@science.unideb.hu (Z.G.); mailod@freemail.hu (R.M.); 2 Centro de Investigacións Científicas Avanzadas (CICA), Departamento de Química, Facultade de Ciencias, Universidade da Coruña, 15071 A Coruña, Spain; rocio.uzal@udc.es (R.U.-V.); david.esteban@udc.es (D.E.-G.); 3 Departamento de Química Inorgánica, Facultad de Ciencias, Universidade de Vigo, As Lagoas, Marcosende, 36310 Pontevedra, Spain; paulo@uvigo.es; 4 Department of Chemistry and Biochemistry, Texas Christian University, 2950 West Bowie Street, Fort Worth, TX 76109, USA; kristof.pota@tcu.edu; 5 Centre de Biophysique Moléculaire, CNRS, UPR 4301, Rue Charles-Sadron, CEDEX 2, 45071 Orléans, France; agnes.pallier@cnrs.fr

**Keywords:** manganese, magnetic resonance imaging, stability, dissociation kinetics, water exchange, contrast agents, macrocycles

## Abstract

We report two macrocyclic ligands based on a 1,7-diaza-12-crown-4 platform functionalized with acetate (***t*O2DO2A^2−^**) or piperidineacetamide (***t*O2DO2AM^Pip^**) pendant arms and a detailed characterization of the corresponding Mn(II) complexes. The X−ray structure of [Mn(***t*O2DO2A**)(H_2_O)]·2H_2_O shows that the metal ion is coordinated by six donor atoms of the macrocyclic ligand and one water molecule, to result in seven-coordination. The Cu(II) analogue presents a distorted octahedral coordination environment. The protonation constants of the ligands and the stability constants of the complexes formed with Mn(II) and other biologically relevant metal ions (Mg(II), Ca(II), Cu(II) and Zn(II)) were determined using potentiometric titrations (*I* = 0.15 M NaCl, T = 25 °C). The conditional stabilities of Mn(II) complexes at pH 7.4 are comparable to those reported for the cyclen-based ***t*DO2A^2−^** ligand. The dissociation of the Mn(II) chelates were investigated by evaluating the rate constants of metal exchange reactions with Cu(II) under acidic conditions (*I* = 0.15 M NaCl, T = 25 °C). Dissociation of the [Mn(***t*O2DO2A**)(H_2_O)] complex occurs through both proton− and metal−assisted pathways, while the [Mn(***t*O2DO2AM^Pip^**)(H_2_O)] analogue dissociates through spontaneous and proton-assisted mechanisms. The Mn(II) complex of ***t*O2DO2A^2−^** is remarkably inert with respect to its dissociation, while the amide analogue is significantly more labile. The presence of a water molecule coordinated to Mn(II) imparts relatively high relaxivities to the complexes. The parameters determining this key property were investigated using ^17^O NMR (Nuclear Magnetic Resonance) transverse relaxation rates and ^1^H nuclear magnetic relaxation dispersion (NMRD) profiles.

## 1. Introduction

The accurate diagnosis of different pathologies using magnetic resonance imaging (MRI) often relies on the use of contrast agents, which are generally small paramagnetic Gd(III) complexes that alter the relaxation times of water proton nuclei in the surroundings of the agent [1,2]. These Gd(III)-based contrast agents (GBCAs) have been routinely used in clinical practice over the last 30 years and were considered safe pharmaceuticals for a long time [3]. However, the safety of GBCAs was questioned after the discovery of a new disease called nephrogenic systemic fibrosis (NSF) in 2006, which occurred in patients with renal impairment and was associated with the administration of contrast agents [4,5]. This prompted the medicine agencies to issue recommendations for the administration of contrast agents, which reduced the number of new NSF cases [6]. More recently, it was discovered that gadolinium accumulates in the brain and other tissues of patients that received GBCAs, though it is not clear whether the metal ion is still coordinated by the ligand in these deposits or the complex dissociated in vivo [7]. Furthermore, no toxicity associated with the presence of Gd deposits has been reported so far [8].

The concerns about toxicity issues prompted bioinorganic chemists to search for safer alternatives that could potentially replace GBCAs in clinical practice [9]. One of the most widely explored alternatives relies on the use of high-spin Mn(II) complexes, which present similar abilities to enhance ^1^H relaxation of water nuclei as analogous Gd(III) complexes [10,11,12]. The main challenge to develop Mn(II)-based contrast agents relies on the difficulties to obtain thermodynamically stable and kinetically inert complexes, to avoid the release of the potentially toxic metal ion [13], while leaving a coordination position available for a water molecule [14]. The inner-sphere water molecule exchanges with bulk water, which provides an efficient mechanism for the relaxation of water ^1^H nuclei in the vicinity of the agent [15].

**Scheme 1:**
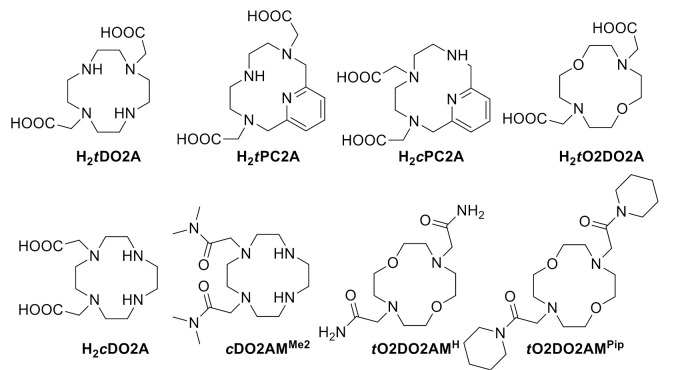
Ligands discussed in the present work.

Different macrocyclic Mn(II) complexes have been investigated as potential contrast agents for MRI applications. The cyclen derivatives ***c*DO2A^2−^** and ***t*DO2A^2−^**(Scheme 1) form relatively stable complexes with Mn(II), but only the complex with ***c*DO2A^2−^**contains a coordinated water molecule [16,17,18]. The incorporation of a rigid pyridyl unit to the macrocyclic unit afforded Mn(II) complexes with a coordinated water molecule, regardless of the arrangement of the acetate groups (***c*PC2A^2−^**or ***t*PC2A^2−^**, Scheme 1) [19,20]. The latter ligands form rather stable and inert complexes with Mn(II) and can be considered promising building blocks for the preparation of contrast agent candidates [21,22]. Herein, we report a detailed study of the Mn(II) complex with ***t*O2DO2A^2−^**, in which the two secondary amine nitrogen atoms of ***t*DO2A^2−^** are replaced by harder oxygen donors. In addition, there is a case in the literature of a 9-membered 1,4,7-triazacyclononane derivative ligand (1,4-diaza-7-oxacyclononane), where the replacement of a nitrogen atom with an oxygen atom in the macrocycle resulted in the formation of seven-coordinate bisaquated Mn(II) complex in solution along with the six-coordinated monoaquated complex [23]. This is in contrast with the structurally related 1,4,7-triazacyclononane ligands, which form six-coordinate complexes in solution with one first-sphere water molecule [24,25]. From this, it can be deduced that as a result of such a donor atom exchange, the seven-coordinate Mn(II) ion becomes somewhat favored in solution (which in turn is expected to contain a coordinated water molecule). This was confirmed recently by J. R. Morrow et al. for a Co(II) complex formed with a bis(amide) derivative ligand (***t*O2DO2AM^H^**) [26]. Furthermore, complexes with amide derivatives of ***c*DO2A^2−^** were found to be considerably more inert than the parent ligand, in spite of the lower thermodynamic stability [27]. Thus, we also report here the ***t*O2DO2AM^Pip^** ligand and the characterization of the corresponding Mn(II) complex. We present the X-ray structures of the Mn(II) complex of ***t*O2DO2A^2−^**, the protonation constants of the ligands and the stability constants of the Mn(II) complexes and those formed with other essential metal ions (Mg(II), Ca(II), Zn(II) and Cu(II)). We also report the dissociation kinetics of the Mn(II) complexes in the presence of Cu(II), and a detailed ^1^H and ^17^O NMR (Nuclear Magnetic Resonance) relaxometric study. The X-ray structure of the Cu(II) complex is also presented.

## 2. Results

### 2.1. Synthesis of the Ligands

The synthesis of H_2_***t*O2DO2A** was achieved by alkylation of the parent crown ether 1,7-diaza-12-crown-4 with *tert*-butyl-2-bromoacetate in acetonitrile in the presence of Na_2_CO_3_ as a base, followed by the hydrolysis of the *tert*-butyl ester groups using trifluoroacetic acid (TFA, see Scheme S1, Supplementary Materials). The ligand was isolated as the trifluoroacetate salt with an overall yield of 58% over the two steps. A similar overall yield (57%) was obtained by Delgado et al. by alkylation with ethyl bromoacetate in ethanol using trimethylamine as a base and subsequent hydrolysis in water [28]. ***t*O2DO2AM^Pip^** was obtained by alkylating the parent macrocycle with 2-bromo-1-(piperidin-1-yl)ethan-1-one, synthesized according to published procedures [29], in the presence of K_2_CO_3_ as a base. The ligand used in the studies was purified by preparative HPLC (High-Performance Liquid Chromatography) on a Luna 10u-Prep C18(2) 100 A (250 × 21.20 mm) column, using MeCN/0.005 M TFA in H_2_O as the eluent.

### 2.2. X-ray Crystal Structures

**Figure 1:**
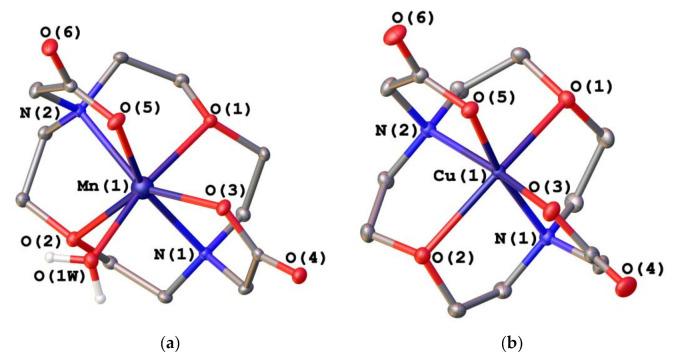
X-ray crystal structures of the [Mn(***t*O2DO2A**)(H_2_O)] (**a**) and [Cu(***t*O2DO2A**)] (**b**) complexes. Non-coordinated water molecules and hydrogen atoms bonded to C atoms are omitted for simplicity. The Oak Ridge Thermal Ellipsoid Plots (ORTEP) are at the 30% probability level.

Slow evaporation of aqueous solutions of the Mn(II) and Cu(II) complexes of ***t*O2DO2A**^2−^ provided single crystals suitable for X-ray diffraction studies. The Mn(II) complex crystallizes in the orthorhombic Pca2_1_ space group, and the asymmetric unit contains two [Mn(***t*O2DO2A**)(H_2_O)] units with slightly different bond distances and angles and four water molecules involved in hydrogen bonds with oxygen atoms of the carboxylate groups and the coordinated water molecules. The Cu(II) analogue crystallized in the monoclinic P2_1_/n space group, with crystals containing the [Cu(***t*O2DO2A**)] complex and hydrate water molecules. Views of the structures of the two complexes are provided in Figure 1, while the bond distances and angles of the metal coordination environments are shown in Table 1. The structure of the Cu(II) complex was reported previously, though the data reported here present a better quality (R1 = 0.0204 vs. 0.063) [30].

The Mn(II) ion in [Mn(***t*O2DO2A**)(H_2_O)] is directly coordinated to the four donor atoms of the macrocyclic unit and two oxygen atoms of the carboxylate pendants. Seven-coordination is completed by an oxygen atom of a coordinated water molecule. The coordination polyhedron can be best described as a capped trigonal prism, as confirmed by performing shape measures with the aid of the SHAPE program (Figure S6, Supplementary Materials) [31,32], which provides shape measures of 0.85, 1.66 and 5.04 for a capped trigonal prism, a capped octahedron and pentagonal bipyramid, respectively [a shape measure S(A) = 0 is expected for a polyhedron fully coincident with the reference polyhedron, with S(A) ranging from 0 to 100]. The upper triangular face of the trigonal prism is defined by N(2), O(1) and O(5), while the lower tripod is delineated by N(1), O(2) and O(1W). The planes defined by these triangular faces are nearly parallel, intersecting at 5.1°. The mean twist angle of the triangular faces is 7.3 ± 1.3°, evidencing a slight distortion of the coordination polyhedron from a trigonal prism (ideal value 0°) towards an octahedron (ideal value 60°). The oxygen atom O(3) is capping the quadrangular face of the prism defined by N(1), O(1), O(5) and O(1W) (rms deviation from planarity 0.101 Å).

The oxygen atoms of the carboxylate groups provide the strongest interaction with the Mn(II) ion, with bond distances falling in the low range observed for seven-coordinate Mn(II) complexes containing acetate groups (*ca.* 2.15–2.30 Å) [18,33,34,35,36]. The oxygen atoms of the crown moiety O(2) presents a considerably longer distance to the metal ion than the second ether O atom [O(1), Table 1]. The conformation adopted by the ligand in the [Mn(***t*O2DO2A**)(H_2_O)] complex is very similar to that determined for the diprotonated [Mn(H_2_**DOTA**)], in which two acetate arms of the ligand in *trans* positions are protonated and remain uncoordinated (DOTA = 1,4,7,10-tetraazacyclododecane-1,4,7,10-tetraacetic acid) [37]. However, the latter complex does not contain a coordinated water molecule, resulting in a six-coordinated Mn(II) ion.

The Cu(II) complex presents a severely distorted octahedral coordination, with two long *trans* distances involving the oxygen atoms of the macrocycle and a *trans* angle that deviates considerably from the ideal 180° [O(2)−Cu(1)−O(1) = 147.99(3)°]. The donor atoms of the equatorial plane defined by N(1), N(2), O(3) and O(5) (rms deviation 0.120 Å) define *cis* angles in the range ca. 84.4–104.1°, and *trans* angles of ca. 169°. The Cu−O distances involving the carboxylate groups are similar to those observed in the six-coordinate Cu(II) complex [Cu(H_2_**DOTA**)] (1.965 Å) [38], while [Cu(H**DO3A**)] shows two significantly different Cu−O distances of 1.923 and 2.077 Å (1,4,7,10-tetraazacyclododecane-1,4,7-triacetic acid) [39].

The macrocyclic unit in [Mn(*t*O2DO2A)(H_2_O)] adopts a (δδδδ) or (λλλλ) conformation, as the two enantiomers are present in the asymmetric unit [40]. This corresponds to a [3333] conformation using Dale’s nomenclature, where the numbers between brackets identify the number of bonds between corners in the macrocyclic ring [41]. This conformation was observed previously in the X-ray structure of a Mn(II) complex formed with a ligand containing the 1,7-diaza-12-crown-4 platform [42], furthermore in complexes with Na(I) [43], Co(II) [26], Ca(II) or Cd(II) [44], as well as the lanthanide ions [45,46]. However, the macrocyclic unit in [Cu(*t*O2DO2A)] adopts a (δλδλ) conformation, which corresponds to a [2424] conformation using Dale’s nomenclature. As a result, the macrocyclic unit is achiral, as it presents two pseudo-mirror planes that bisect the macrocyclic unit and contain either the ether oxygen atoms or the amine nitrogen atoms. A [2424] conformation was also reported for the protonated [Cu(H_2_DOTA)] complex [39]. This conformation is in contrast with the asymmetric (δλλδ) conformation observed for a Cu(II) complex containing the 1,7-diaza-12-crown-4 motif [47].

### 2.3. Protonation Constants of the Ligands

The protonation constants of the ***t*O2DO2A^2^**^−^ and ***t*O2DO2AM^Pip^** ligands were determined by pH-potentiometric titrations with an ionic strength fixed at *I* = 0.15 M NaCl. The results are compared in Table 2 with the equilibrium constants reported for ***t*DO2A^2−^** [17,18] and ***t*PC2A**^2**−**^ [19,48] as well as those measured previously for ***t*O2DO2A^2−^** using 0.1 M Me_4_NNO_3_ as the background electrolyte [49]. Both ***t*O2DO2A^2−^** and ***t*O2DO2AM^Pip^** present two protonation constants with log*K*^H^ values > 6.0, associated to the protonation of the amine N atoms of the ligand. The third protonation constant determined for ***t*O2DO2A^2−^** can be attributed to the protonation of one of the carboxylate groups of the ligand. The log*K*_1_^H^ value determined in 0.15 M NaCl is ~1.5 log units is lower than that determined previously in 0.1 M Me_4_NNO_3_, while the remaining protonation constants do not change significantly with ionic strength. This indicates that the ***t*O2DO2A^2−^** ligand forms a relatively stable complex with Na(I), as observed previously for different cyclen-based ligands [50,51]. Noteworthy, this effect is not observed in the cases of ***t*DO2A^2−^** and ***t*PC2A^2−^** (Table 2). The protonation constants determined for ***t*O2DO2AM^Pip^** are lower than those of ***t*O2DO2A^2−^**, in line with the decreased basicity observed previously for amine N atoms when carboxylate pendant groups are replaced by amides [52,53]. The basicity of the 1,7-diaza-12−crown-4 derivative ***t*O2DO2A^2−^** is considerably lower than that of the cyclen and pyclen analogues ***t*DO2A^2−^** and ***t*PC2A^2−^**, in agreement with previous studies [54,55]. The overall basicity of this series of structurally related ligands follows the trend ***t*DO2A^2−^** > ***t*PC2A^2−^** > ***t*O2DO2A^2−^**.

### 2.4. Stability Constants of Metal Complexes

The stability constant of the Mn(II) complexes formed with ***t*O2DO2A^2−^** and ***t*O2DO2AM^Pip^** were determined by pH-potentiometry in 0.15 M NaCl. Furthermore, we also measured the stability constants of the ligands with other metal ions present in vivo (Mg(II), Ca(II), Cu(II) and Zn(II)). The results are presented in Table 3, together with the data reported in the literature for ***t*DO2A^2−^** and ***t*PC2A^2−^** [17,55]. The stability constants determined for ***t*O2DO2A^2−^** using a 0.15 M NaCl background electrolyte are ca. 1.5 log units lower than those reported in 0.1 M Me_4_NNO_3_ [49], which can be ascribed to the formation of a relatively stable complex with Na(I). The stability constants of the complexes formed with ***t*O2DO2AM^Pip^** are lower than those with ***t*O2DO2A^2−^**, as generally observed when negatively charged donor groups are replaced by charge neutral amide pendants [27]. A comparison of the stability constants of the complexes with ***t*O2DO2A^2−^** and ***t*DO2A^2−^** evidences a dramatic drop of the stability when two N atoms of the cyclen scaffold are replaced by oxygen atoms. In the specific case of Mn(II), the stability constant determined for the complex with ***t*DO2A^2−^** is more than five orders of magnitude higher than that of the ***t*O2DO2A^2−^** analogue (Table 3). The stability of the Mn(II) complex with ***t*PC2A^2−^** is even higher. The equilibrium constants log*K*_ML_ determined for the Ca(II) and Zn(II) complexes follow the same trend ***t*PC2A^2−^** > ***t*DO2A^2−^** > ***t*O2DO2A^2−^** > ***t*O2DO2AM^Pip^**.

A more meaningful comparison of the stabilities of metal complexes formed with ligands with different basicity is provided by the pM values, which in the case of Mn(II) complexes is often defined as pMn = −log[Mn]_free_, with *c*_L_ = *c*_Mn_ = 10^−5^ M (pH = 7.4) [56]. The complexes of ***t*O2DO2A^2−^** and ***t*DO2A^2−^** are characterized by similar pMn values, in spite of the higher log*K*_MnL_ values determined for the latter. This is related to the high basicity of the ***t*DO2A^2−^** ligand, which results in a more efficient proton competition with the metal. The pMn value calculated for the complex with ***t*O2DO2AM^Pip^** is only slightly lower than those characterizing the complexes with ***t*O2DO2A^2−^** and ***t*DO2A^2−^**. However, the ***t*PC2A^2−^** ligand clearly forms the most stable complex with Mn(II) among this series of structurally related macrocycles.

**Figure 2:**
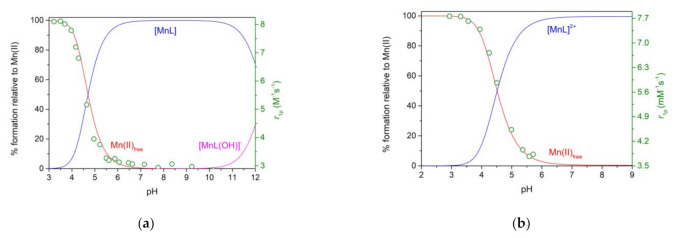
Species distribution diagrams of the Mn(II) − L − H^+^ systems ([Mn(II)] = [L] = 1.0 mM, solid lines, L = ***t*O2DO2A^2−^** (**a**) or ***t*O2DO2AM^Pip^** (**b**) and the normalized *r*_1p_ relaxivity values obtained as function of pH at 20 MHz, T = 25 °C and *I* = 0.15 M NaCl.

The stability constants determined by pH-potentiometric titrations were further verified by measuring the pH-dependency of the longitudinal ^1^H relaxivities of equimolar mixtures of Mn(II) and the corresponding ligand. The relaxivities of the solutions increase below pH ~7, reaching a maximum at about pH 3. The relaxivity observed at low pH (~8 mM^−1^ s^−1^) is close to that observed for the aqua-complex [Mn(H_2_O)_6_]^2+^ at the same magnetic field strength and temperature (0.49 T, 25 °C). This increase in relaxivity correlates with the dissociation of the complexes predicted by the protonation and stability constants reported in Table 3, as shown by the corresponding speciation diagrams (Figure 2).

### 2.5. Dissociation Kinetics

**Figure 3:**
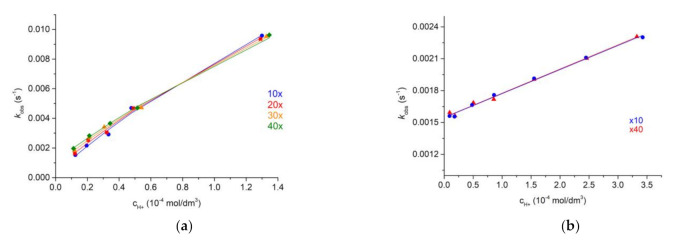
Dissociation rates (*k*_obs_) of the Mn(II) complexes formed with ***t*O2DO2A^2−^** (**a**) or ***t*O2DO2AM^Pip^** (**b**) obtained in the presence of excess Cu(II) and plotted as a function of H^+^ ion concentration (*I* = 0.15 M NaCl, T = 25 °C).

The inertness of the Mn(II) complexes of ***t*O2DO2A^2−^** and ***t*O2DO2AM^Pip^** with respect to their dissociation was investigated by evaluating the rate constants of metal exchange reactions with Cu(II) (an abundant essential metal ion which offers an easy tool of following the course of the dissociation) in the pH-range of 3.8–4.9 and 3.4–5.0, respectively. The rate constants (*k*_obs_) increase with increasing H^+^ ion concentration (Figure 3). This dependence on [H^+^] shows a saturation behavior for ***t*O2DO2A^2−^**, while it is linear for the amide derivative (***t*O2DO2AM^Pip^**). Furthermore, the *k*_obs_ values determined for the dissociation of the ***t*O2DO2A^2−^** complex increase with increasing Cu(II) concentration. Thus, the Mn(II) complex of ***t*O2DO2A^2−^** dissociates by following proton− and metal−assisted pathways (Scheme S1). The rate constants measured for the ***t*O2DO2AM^Pip^** complex are not affected by the concentration of Cu(II). Furthermore, the linear dependence of *k*_obs_ with [H^+^] is characterized by a rather large value of the intercept with the y axis, which evidences that dissociation proceeds through both spontaneous and proton-assisted mechanisms. The dissociation pathways followed by these complexes can be accounted for by Equation (1), where [Mn(L)]_t_ is the total concentration of the Mn(II) complex, the spontaneous dissociation is characterized by *k*_0_, the proton-assisted pathway is associated to *k*^H^ and *k*_H_^H^, and the metal-assisted dissociation is represented by *k*_M_.

(1) $$-\frac{d\left[\mathrm{Mn}\left(L\right)\right]}{dt}\;=\;k_{\mathrm{obs}}{\left[\mathrm{Mn}\left(L\right)\right]}_{t}\;=\;k_{0}\left[\mathrm{Mn}\left(L\right)\right]\;+\;k_{H}\left[\mathrm{Mn}\left(\mathrm{HL}\right)\right]\;+\;k_{H}^{H}\left[\mathrm{Mn}\left(H_{2}L\right)\right]\;+\;k_{M}\left[\mathrm{Mn}\left(L\right)\mathrm{Cu}\right]$$

Using the expressions of the equilibrium constants for the stability and protonation constants of the complexes (both mono and diprotonated species) and the stability constant for the dinuclear intermediate [Mn(L)Cu], one can derive Equation (2):

(2) $$k_{obs}\;=\;\frac{k_{0}\;+\;k_{1}\left[H^{+}\right]\;+\;k_{2}{\left[H^{+}\right]}^{2}\;+\;k_{3}\left[{\mathrm{Cu}}^{2+}\right]}{1\;+\;K_{\mathrm{Mn}\left(\mathrm{HL}\right)}\left[H^{+}\right]\;+\;K_{\mathrm{Mn}\left(\mathrm{HL}\right)}K_{\mathrm{Mn}\left(H_{2}L\right)}{\left[H^{+}\right]}^{2}\;+\;K_{\mathrm{Mn}\left(L\right)\mathrm{Cu}}\left[{\mathrm{Cu}}^{2+}\right]}.$$

In this expression *k*_1_ = *k*_H_ × *K*_Mn(HL)_, *k*_2_ = *k*_H_^H^ × *K*_Mn(HL)_ × *K*_Mn(H2L)_ and *k*_3_ = *k*_M_ × *K*_Mn(L)Cu_. However, Equation (2) can be further simplified to fit the experimental *k*_obs_ values depending on the pathways that play a significant role for the specific system under investigation. For the ***t*O2DO2A^2−^** complex the data could be satisfactorily fitted by using *k*_1_, *k*_3_, *K*_Mn(HL)_ and *K*_Mn(L)Cu_ as fitting parameters. This indicates that the spontaneous dissociation and the acid-assisted dissociation involving a diprotonated intermediate, characterized by *k*_0_ and *k*_2_, respectively, play a negligible role in the dissociation of the complex under the experimental conditions used for kinetic studies. For the ***t*O2DO2AM^Pip^** complex the kinetic data were successfully fitted using *k*_0_ and *k*_1_ as fitting parameters (Table 4).

The rate constant characterizing the proton assisted dissociation of the ***t*O2DO2A^2−^** complex is comparable to those of the ***t*O2DO2A^2−^** and ***t*PC2A^2−^** derivatives. The Cu(II)-assisted mechanism does not contribute to the dissociation of the ***t*DO2A^2−^** complex but becomes relevant for ***t*PC2A^2−^** and ***t*O2DO2A^2−^**. The latter complex is characterized by a *k*_3_ constant about 2.5 times higher than the ***t*PC2A^2−^** analogue. The half-lives estimated for these three charge-neutral Mn(II) complexes do not however differ significantly. The proton-assisted dissociation is less efficient at promoting the dissociation of the complex with ***t*O2DO2AM^Pip^**, as expected due to its positive charge. A similar observation was evidenced by A. Forgács and co-workers when comparing the dissociation kinetic data of the Mn(II) complexes formed with ***c*DO2A^2^^−^** and its bis(methyl)amide derivative (***c*DO2AM^Me2^**) ligands [27]. However, the spontaneous dissociation, presumably related to the weak binding of the ligand evidenced by the low stability constant, provides a very efficient path for complex dissociation. As a result, the Mn(II) complex with ***t*O2DO2AM^Pip^** is very labile, as reflected in the very short lifetime calculated at pH 7.4 in the presence of 0.01 mM Cu(II) (Table 4).

### 2.6. Hydration Numbers

The efficiency of a *T*_1_ contrast agent is generally assessed in vitro by measuring its proton relaxivity *r*_1p_, which determines the ability of a 1 mM solution of the paramagnetic complex to accelerate the relaxation time of solvent water proton nuclei. The inner-sphere contribution to relaxivity is proportional to the number of water molecules coordinated to the paramagnetic ion *q*. The *q* values of Mn(II) complexes can be estimated by measuring transverse ^17^O NMR relaxivities, following the methodology proposed by Gale [57]. This method relates the hydration number *q* with the transverse ^17^O relaxivity at the maximum of the plot versus inverse temperature (*r*_2*p,max*_) according to: Equation (3):

(3) $$q\;≅\;\frac{r_{2p,max}}{510}.\;$$

**Figure 4:**
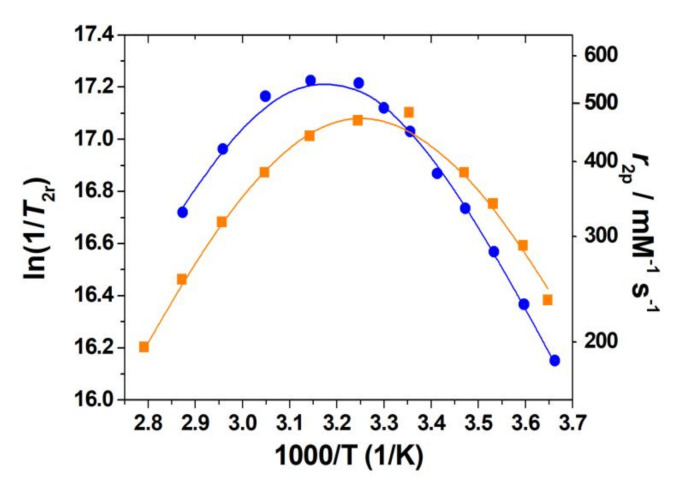
Reduced transverse ^17^O relaxation rates for the Mn(II) complexes of ***t*O2DO2A^2−^** (orange squares) and ***t*O2DO2AM^Pip^** (blue circles) and transverse relaxivities (right *y* axis) measured at 9.4 T. The solid lines correspond to the fit of the data as described in the text.

The ^17^O relaxivities measured for the Mn(II) complexes of ***t*O2DO2A^2−^** and ***t*O2DO2AM^Pip^** (Figure 4) show *r*_2p,max_ of 480 and 556 mM^−1^ s^−1^, respectively, which correspond to *q* values of 0.94 and 1.09. This points to the presence of a water molecule coordinated to the metal ion in both complexes, in agreement with the X-ray crystal structure of the Mn(II) complex described above.

**Figure 5:**
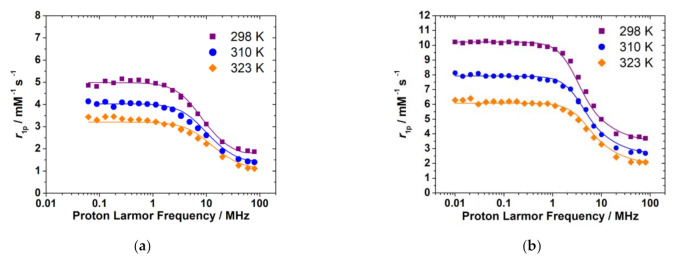
^1^H nuclear magnetic relaxation dispersion (NMRD) profiles recorded at different temperatures for the Mn(II) complexes of ***t*O2DO2A^2−^** ((**a**), pH 7.4) and ***t*O2DO2AM^Pip^** ((**b**), pH 7.4). The solid lines correspond to the fits of the data as described in the text.

The hydration number of Mn(II) complexes can also be estimated by measuring ^1^H nuclear magnetic relaxation dispersion (NMRD) profiles, which are plots of the longitudinal ^1^H relaxivity *r*_1p_ as a function of the proton Larmor frequency (on a logarithmic scale, Figure 5). The relaxivity observed at low field (i.e., 0.1 MHz) was related with the hydration number using Equation (4), where *FW* is the molecular weight [58].

(4) $$q\;=\;\frac{r_{1p}}{9.16\left\{1\;-\;e^{-0.00297\times FW}\right\}}.$$

The ^1^H relaxivities measured for the Mn(II) complexes of ***t*O2DO2A^2−^** and ***t*O2DO2AM^Pip^** at 0.1 MHz (25 °C) are 4.96 and 10.15 mM^−1^ s^−1^, respectively, which correspond to *q* values of 0.85 and 1.46. Thus, this method provides hydration numbers in reasonable agreement with those estimated from ^17^O transverse relaxivities. Nevertheless, the results obtained with the two methods confirm that the studied Mn(II) complexes are monohydrated.

### 2.7. Water Exchange

The relaxivities of Mn(II) complexes depend upon a relatively large number of microscopic parameters and their detailed analysis requires the use of complementary techniques. For instance, the exchange rate of coordinated water molecules (*k*_ex_) can be accurately determined using transverse ^17^O NMR measurements. The reduced transverse ^17^O NMR relaxation rates determined for the Mn(II) complexes ***t*O2DO2A^2−^** and ***t*O2DO2AM^Pip^** show a maximum that signals the changeover from slow exchange at low temperatures to the fast exchange regime at high temperatures (Figure 4). This maximum is shifted to higher temperatures for the complex with ***t*O2DO2AM^Pip^**, which anticipates a slower water exchange than for the ***t*O2DO2A^2−^** analogue.

The ^17^O NMR data recorded for the Mn(II) complex of ***t*O2DO2A^2−^** was analyzed using the Swift-Connick equations [59,60]. The results of the fit yielded the water exchange rate at 298 K, *k*_ex_^298^, and its activation enthalpy, Δ*H*^‡^, the scalar hyperfine coupling constant, *A*_O_/*ħ*, and the longitudinal relaxation rate of the electron spin, 1/*T*_1_*e*^298^. We assumed an Arrhenius behavior of 1/*T*_1_*e*^298^ with temperature, with a small activation energy of 1 kJ mol^−1^. The results of the fit afforded a large standard deviation for 1/*T*_1_*e*^298^ but provided small standard deviations for the remaining parameters. The value of the hyperfine coupling constant *A*_O_/*ħ* obtained from the fit of the data falls in the upper range observed for Mn(II) complexes (*ca*. (−30 to −45) × 10^6^ rad s^−1^) [16,57]. In a previous work, we showed that the *A*_O_/*ħ* values can be accurately calculated using Density Functional Theory (DFT) [61]. Thus, we used DFT calculations at the M11/Def2−TZVP level (see computational details below) to estimate the value of *A*_O_/*ħ*, which was found to be −45.5 × 10^6^ rad s^−1^ (−7.24 MHz). This value is in excellent agreement with that obtained from the analysis of the ^17^O NMR data (Table 5). Attempts to fit the ^17^O NMR data of the ***t*O2DO2AM^Pip^** complex by varying the four parameters were unsuccessful. We thus calculated *A*_O_/*ħ* using DFT (−40.1 × 10^6^ rad s^−1^) and fixed its value during the fitting procedure. The similar *A_O_/*ħ** values calculated for the two complexes appear to be related to the very similar bond distances involving the coordinated water molecule (2.85 Å in the DFT structures).

The water exchange rate determined for the ***t*O2DO2AM^Pip^** complex is ~1.5 times lower than for the ***t*O2DO2A^2−^** analogue. This follows the general trend observed for seven-coordinate Mn(II) complexes, for which water exchange decreases on increasing the positive charge of the complex (i.e., complexes of ***c*DO2A^2−^** and its bis-amides) [27]. This effect is however not very pronounced for the ***t*O2DO2AM^Pip^** and ***t*O2DO2A^2−^** complexes. Water exchange in seven-coordinate Mn(II) complexes is likely to follow dissociatively activated mechanisms involving six-coordinate transition states. Increasing the positive charge of the complex results in a stronger Mn−water interaction and thus in slower exchange. The assessment of the reaction mechanism requires variable pressure measurements, which provide access to activation volumes. However, this type of experiments can be performed only in a few laboratories [62]. Nevertheless, the activation entropy, Δ*S*^‡^, can also provide valuable mechanistic insight [63]. For the ***t*O2DO2A^2−^** complex we obtained a small positive value for Δ*S*^‡^, while for the ***t*O2DO2AM^Pip^** complex Δ*S*^‡^ has a small negative value, suggesting an interchange mechanism for the water exchange reactions. Thus, the Δ*S*^‡^ values suggest that the entering water molecule is involved in the transition state responsible for the water exchange mechanism, and thus increasing the positive charge of the complex has a minor effect in the water exchange rate.

The water exchange in the aqua-complex [Mn(H_2_O)_6_]^2+^ is relatively slow (28.2 × 10^6^ s^−1^), and it is generally accelerated upon complexation with chelating ligands [64]. The *k*_ex_^298^ values determined for the ***t*O2DO2AM^Pip^** and ***t*O2DO2A^2−^** complexes are close to that of the aqua-complex and can thus be considered rather low. Similar exchange rates were determined for seven coordinate Mn(II) complexes with pentadentate macrocycles [56], while *k*_ex_^298^ values as high as 6 × 10^8^ s^−1^ have been reported [16]. The water exchange rate determined for the ***t*O2DO2A^2−^** complex is lower than that of the ***t*PC2A^2−^** complex, while the ***t*DO2A^2−^** analogue lacks the inner-sphere water molecule. These results indicate that the complex with ***t*O2DO2A^2−^** provides the most open structure among this series of structurally related ligands, allowing a rather strong coordination of the water ligand.

### 2.8. Analysis of the ^1^H NMRD Profiles

The NMRD profiles presented in Figure 5 were analyzed by using the Solomon-Bloembergen-Morgan theory of paramagnetic relaxation to account for the inner-sphere contribution to relaxivity, and Freed’s model to analyze the outer-sphere contribution to relaxivity [65]. Given the large number of parameters influencing *r*_1p_ we fixed some of them to reasonable values: The distance between the water protons of the coordinated water molecule and the Mn(II) ion was fixed to 2.85 Å for the ***t*O2DO2A^2−^** and ***t*O2DO2AM^Pip^** complexes, the relative diffusion coefficient *D*_MnH_^298^, its activation energy *E*_MnH_ and the distance of closest approach of an outer-sphere water molecules *a*_MnH_ were fixed to common values of 23 × 10^−10^ m^2^ s^−1^, 18 kJ mol^−1^ and 3.6 Å. The parameters characterizing water exchange (*k*_ex_^298^ and Δ*H*^‡^) were fixed to the values obtained from ^17^O NMR measurements. The results of the fit provided the rotational correlation time *τ*_R_ and its activation energy *E*_R_, the mean square zero-field splitting energy Δ^2^, the correlation time for the modulation of the zero-field-splitting interaction, *τ*_V,_ and its activation energy *E*_v_. For the latter, a value of 1 kJ mol^−1^ was assumed. The parameters calculated are shown in Table 5.

The *τ*_R_ values obtained for the two complexes are very reasonable considering their molecular weights. The higher relaxivity observed for the ***t*O2DO2AM^Pip^** complex at high field (>10 MHz) is mainly related to a longer *τ*_R_ value, associated with the increased size of the complex.

## 3. Materials and Methods

### 3.1. General Methods

Elemental analyses were obtained using a Carlo Erba 1108 elemental analyzer (Valencia, CA, USA). Electrospray-Ionization Time-of-Fligth (ESI-TOF) mass spectra were recorded using a LC-Q-q-TOF Applied Biosystems QSTAR Elite spectrometer (Waltham, MA USA), working in the positive mode. ^1^H and ^13^C NMR spectra were recorded at 25 °C on Bruker Avance 360 and 500 MHz spectrometers (Bruker, Billerica, MA, USA).

#### Reagents

All reagents were obtained from commercial sources and were used as received. The 1,7-diaza-12-crown-4 macrocycle was purchased from the CheMatech (Dijon, France) whereas the 2-bromo-1-(piperidin-1-yl)ethan-1-one was synthesized by following the published procedure [29]. Solvents were of reagent grade and were used without further purification.

### 3.2. Synthesis

**Di-tert-butyl 2,2′-**(**1,7-dioxa-4,10-diazacyclododecane-4,10-diyl**)**diacetate** (**1**). A mixture of 1,7-diaza-12-crown-4 (1.00 g, 5.74 mmol) and Na_2_CO_3_ (4.87 g, 45.9 mmol) in acetonitrile (150 mL) was stirred for 30 min and then tert-butyl-2-bromoacetate (2.35 g, 12.1 mmol) and a catalytic amount of KI were added. The mixture was stirred at 45 °C under an inert atmosphere (Ar) for a period of 120 h and then the excess Na_2_CO_3_ was filtered off. The filtrate was concentrated to dryness and the yellow oil was extracted with a 1:3 mixture of H_2_O and CH_2_Cl_2_ (300 mL). The organic phase was evaporated to dryness to give an oily residue that was purified by preparative medium pressure liquid chromatography (neutral Al_2_O_3_ with a CH_2_Cl_2_/MeOH mixture as the eluent; gradient 0–10%) to give **1** as a white solid (1.66 g, 72%). δ_H_ (CDCl_3_, 500 MHz, 298 K): 3.57 (t, 8H, ^3^*J* = 4.7 Hz), 3.37 (s, 4H), 2.92 (t, 8H, ^3^*J* = 4.7 Hz), 1.45 ppm (s, 18H). δ_C_ (CDCl_3_, 125.8 MHz, 298 K): 171.2, 80.9, 69.6, 57.5, 54.6, 28.2 ppm. MS-ESI^+^, *m/z*: 403 ([C_20_H_39_N_2_O_6_]^+^). Elem Anal. Calcd. for C_20_H_38_N_2_O_6_: C 59.68, H 9.52, N 6.96%. Found: C 59.43, H 9.47, N 6.76%. IR (ATR): 1723 cm^−1^ (C = O).

**2,2′-**(**1,7-Dioxa-4,10-diazacyclododecane-4,10-diyl**)**diacetic acid** (**H_2_*t*O2DO2A**). The di-tert-butyl ester (**1**) (1.66 g, 4.12 mmol) was dissolved in a 1:1 mixture of dichloromethane and trifluoroacetic acid (10 mL). The mixture was heated to reflux with stirring for 24 h, and then the solvents were removed in a rotary evaporator to yield a yellow oil. This was dissolved in H_2_O (10 mL) and the solvent was evaporated. This process was repeated twice, and then three times with diethyl ether. The white solid was dried under vacuum (1.77 g, 80%). δ_H_ (D_2_O, pD = 7.0, 500 MHz, 298 K): 3.78 (t, 8H, ^3^*J* = 4.9 Hz), 3.75 (s, 4H), 3.45 ppm (t, 8H, ^3^*J* = 4.9 Hz). δ_C_ (D_2_O, pD = 7.0, 125.8 MHz, 298 K): 171.1, 64.9, 58.4, 55.0 ppm. MS-ESI^+^, *m/z*: 291 ([C_12_H_23_N_2_O_6_]^+^). Elem Anal. Calcd. for C_12_H_22_N_2_O_6_·2CF_3_COOH·H_2_O: C 35.83, H 4.89, N 5.22%. Found: C 35.71, H 4.66, N 4.92%. IR (ATR): 1740, 1697 and 1651 cm^−1^ (C = O).

**2,2′-**(**1,7-dioxa-4,10-diazacyclododecane-4,10-diyl**)**bis**(**1-**(**piperidin-1-yl**)**ethan-1-one**) (***t*O2DO2AM^Pip^**). A solution of 1,7-diaza-12-crown-4 (0.50 g, 2.87 mmol), synthesized 2−bromo-1-(piperidin-1-yl)ethan-1-one (1.24 g, 6.03 mmol) and K_2_CO_3_ (1.38 g, 10.0 mmol) in anhydrous acetonitrile was heated to 65 °C with stirring for 3 days. The solution was then filtered and the filtrate evaporated under vacuum. The resulting oily residue was redissolved in a mixture of water and acetonitrile (50:50 by volume) and purified by preparative HPLC using a Luna 10u-Prep C18(2) 100A (250 × 21.20 mm) column and MeCN:H_2_O/TFA was applied as eluent (the TFA was present only in water at 0.005 M concentration). The product was collected as yellowish solid by repeated freeze-drying using 1.0 M HCl. Yield: 0.76 g (45%). δ_H_ (D_2_O, pD = 2.0, 360.13 MHz, 298 K): 4.26 (s, 4H), 3.63 (bm, 8H), 3.43, 3.33 (bm, 8H), 3.21, 3.06 (bm, 8H), 1.36, (bm, 8H), 1.27 ppm (m, 4H). δ_C_ (D_2_O, pD = 2.0, 90.56 MHz, 298 K): 162.36, 64.28, 57.65, 56.33, 46.00, 43.96, 25.53, 24.98, 23.40 ppm. MS-ESI^+^, *m/z*: 425.83 ([C_22_H_41_N_4_O_4_]^+^) and 447.50 ([C_22_H_40_N_4_O_4_Na]^+^). Elem Anal. Calcd. for C_22_H_40_N_4_O_4_·3HCl·3H_2_O: C 44.97, H 8.92, N 9.54%. Found: C 44.94, H 8.40, N 9.53%.

**Preparation of the [M**(***t*O2DO2A**)**]·*x*H_2_O complexes. General Procedure.** A solution of *t*O2DO2A ·2CF_3_COOH·H_2_O (0.095 g, 0.178 mmol), triethylamine (0.072 g, 0.711 mmol) and Cu(OTf)_2_ or Mn(NO_3_)_2_·4H_2_O (0.178 mmol) in a mixture of 2-propanol and MeOH (9:1, 10 mL) was heated to reflux for 4 h. The reaction was allowed to cool to room temperature and was then concentrated to dryness. The addition of 5 mL of THF resulted in the formation of a precipitate, which was isolated by filtration. The solid was then suspended in 10 mL of THF and stirred at room temperature for 24 h. The solid was isolated by filtration, washed with THF and diethyl ether and dried under vacuum.

**[Cu**(***t*O2DO2A**)**]·2H_2_O**. Light blue solid. Yield 0.047 g, 65%. Elem Anal. Calcd. for C_12_H_20_CuN_2_O_6_·2H_2_O: C, 37.16; H, 6.24; N, 7.22%. Found: C, 36.88; H, 6.04; N, 7.14%. HR-MS (ESI+, MeOH:CH_3_CN:H_2_O 9:1:1): m/z 352.0696; calcd. for [C_12_H_21_CuN_2_O_6_]+ 352.0690. IR (ATR, cm^−1^): 1607 (C = O). Single crystals with formula **[Cu**(***t*O2DO2A**)**]·2H_2_O** were obtained by slow evaporation of an aqueous solution of the complex.

**[Mn**(***t*O2DO2A**)**]·2.5H_2_O**. White solid. Yield 0.013 g, 39%. Elem Anal. Calcd. for C_12_H_20_CuN_2_O_6_·2.5H_2_O: C, 36.32; H, 6.35; N, 7.06%. Found: C, 36.19; H, 6.50; N, 7.07%.HR-MS (ESI^+^, MeOH:CH_3_CN:H_2_O 9:1:1): *m*/*z* 366.0599; calcd. for [C_12_H_20_MnN_2_NaO_6_]^+^ 366.0594; *m*/*z* 709.1309; calcd. for [C_24_H_40_Mn_2_N_4_NaO_12_]^+^ 709.1296; *m*/*z* 1052.2038; calcd. for [C_36_H_60_Mn_3_N_6_NaO_18_]^+^ 1052.1998. IR (ATR, cm^−1^): 1609, 1575 (C = O). Single crystals with formula **[Mn**(***t*O2DO2A**)(**H_2_O**)**]·2H_2_O** were obtained by the slow evaporation of an aqueous solution of the complex.

### 3.3. Equilibrium Studies

The protonation constants of the ligands as well as the protonation and stability constants of their complexes formed with essential metal ions (Mg(II), Ca(II), Mn(II), Zn(II) and Cu(II)) were determined by pH-potentiometry in the presence of 0.15 M NaCl to mimic the in vivo circumstances. The potentiometric titrations were performed in 6 mL volume under N_2_ atmosphere (to avoid the effect of CO_2_) and the temperature was set to 25.0 ± 0.1 °C using a circulating water bath. The pH values were measured with a combined pH glass electrode (Metrohm, filled with 3 M KCl) and the automated titrations were carried out by a 702 SM titrino system (Metrohm). The 0.1 M HCl and NaOH solutions were prepared from Fisher Chemicals concentrates. The electrode was calibrated by titrating an HCl solution with 0.1 NaOH in 0.15 M electrolyte solution [66]. The electrode standard potential (E°) and the slope factor (f) were calculated by plotting the potential-p[H] data pairs. The concentration of the ligand and metal ion was generally 2 mM in the titrations. For the calculation of the protonation and stability constants 70–140 mL NaOH–potential data pairs obtained in the pH range of 1.7–11.5 (310–(−270) mV) were used. By fitting the data to Equation (5), the protonation constants of the ligand were calculated.

(5) $$K_{i}^{H}\;=\;\frac{{[H}_{i}L]}{{[H}_{i-1}L]{\left[H\right]}^{+}}\;\;i=\;1–3.$$

For the solution equilibria of the complexes, the formation of [MHL], [ML] and [M(OH)L] species were assumed (defined by Equations (6)–(8)).

(6) $$K_{\mathrm{ML}\;}=\;\frac{\left[\mathrm{ML}\right]}{\left[M\right]\left[L\right]}$$

(7) $$K_{\mathrm{ML}}^{H}\;=\;\frac{\left[\mathrm{MHL}\right]}{\left[\mathrm{ML}\right]{[H}^{+}]}$$

(8) $$K_{\mathrm{ML}}^{\mathrm{OH}}\;=\;\frac{{[M(\mathrm{OH})L][H}^{+}]}{\left[\mathrm{ML}\right]}.$$

In the calculations, the value of *K*_w_ (ion product of water) was set to 13.78 corresponding to 0.15 M NaCl ionic strength. The PSEQUAD program was used to perform the equilibrium calculations [67].

### 3.4. Kinetic Studies

The inertness of the [Mn(***t*O2DO2A**)] and [Mn(***t*O2DO2A^Pip^**)]^2+^ complexes was studied by metal exchange reactions using Cu(II) ion as ligand scavenger in 10–40-fold excess relative to the complex concentration (c_complex_ = 0.4 mM). The reactions were monitored by a JASCO V750 UV-Vis spectrophotometer (Waltham, MA, USA) (25 °C (Peltier thermostated), *I* = 0.15 M NaCl, 1 cm micro quartz cuvette—V_tot_ = 500 μL). The kinetic studies were carried out in *N,N*′-dimethylpiperazine buffer to maintain the pH constant in the range 3.4–5.0 (c_DMP_ = 50 mM, log *K*_2_^H^ = 4.19). The rate constants (*k*_obs_) of the reactions were calculated by fitting the absorbance-time data pairs to the Equation (9).

(9) $$A_{t}\;=\;\left(A_{0}-A_{t}\right)e^{k_{obs}t}+A_{e},$$

where *A*_*t*_, *A*_0_ and *A*_*e*_ are the absorbance at time t, at the start and at equilibrium, respectively. The high excess of the Cu(II) ion ensures the pseudo-first-order condition for the reactions, thus the reaction rate can be given by Equation (10) and *k*_obs_ can be handled as pseudo-first-order rate constant.

(10) $$-\frac{d{\left[\mathrm{MnL}\right]}_{t}}{dt}\;=\;k_{obs}{\left[\mathrm{MnL}\right]}_{t}.$$

The calculations were performed with the computer program Micromath Scientist, version 2.0 (Salt Lake City, UT, USA) by using a standard least-squares procedure.

#### H- and ^17^O Relaxometry

The ^1^H-NMRD measurements were carried out by using a Stelar SMARTracer Fast Field Cycling relaxometer (Stelar, Pavia, Italy) (0.01–10 MHz) and a Bruker WP80 NMR electromagnet (Bruker, Billerica, MA, USA) adapted to variable field measurements (20–80 MHz) controlled by a SMARTracer PC-NMR console (Stelar, Pavia, Italy). The NMRD profiles of the Mn(II) complexes (c_complex_ = 1.00 mM) were recorded in aqueous solution at three different temperatures (25, 37 and 50 °C) in the presence of HEPES (4-(2-hydroxyethyl)-1-piperazineethanesulfonic acid) buffer (40 mM, pH = 7.4) to maintain the pH. The temperature of the samples was managed by a VTC91 temperature control unit (calibrated by a Pt resistance temperature probe) and maintained by gas flow.

^17^O NMR measurements were performed on an aqueous solution of the Mn(II) complexes (pH = 7.4, 1.0 mM) and of a diamagnetic reference (HClO_4_ acidified water, pH = 3.3) in order determine the longitudinal (1/*T*_1_) and transverse (1/*T*_2_) relaxation rates and chemical shifts in the temperature range 273–348 K using a Bruker Avance 400 (9.4 T, 54.2 MHz) spectrometer (Bruker, Billerica, MA, USA). The temperature values were calculated according to the standard calibration by means of ethylene glycol and methanol as standards [68]. For the determination of the 1/*T*_1_ and 1/*T*_2_ relaxation rates, the inversion−recovery and Carr−Purcell−Meiboom−Gill (CPMG) spin−echo techniques were used [69]. To avoid susceptibility corrections of the chemical shifts, a glass sphere fitted into a 10 mm NMR tube was used to contain the samples. ^17^O enriched water (10% H_2_^17^O, CortecNet) was added to the samples reaching around 2% enrichment to increase the sensitivity.

The least-squares fit of the ^17^O NMR and of the NMRD data was performed using Visualiseur/Optimiseur running on a MATLAB 8.3.0 (R2014a) platform [70].

### 3.5. X-ray Diffraction Studies

Single crystals of [Cu(***t*O2DO2A**)]·2H_2_O and [Mn(***t*O2DO2A**)(H_2_O)]·2H_2_O were analyzed by X-ray diffraction; a summary of the crystallographic data and the structure refinement parameters is reported in Table 6. Crystallographic data were collected at 100 K using a Bruker D8 Venture diffractometer (Bruker, Billerica, MA, USA) with a Photon 100 CMOS detector (Bruker Daltonics, Billerica, MA, USA) and Mo-Kα radiation (λ = 0.71073 Å) generated by an Incoatec high brilliance microfocus source equipped with Incoatec Helios multilayer optics (Incoatec GmbH, Geesthacht, Germany). The software APEX3 [71] was used for collecting frames of data, indexing reflections and the determination of lattice parameters, SAINT [72] for the integration of intensity of reflections and SADABS [73] for scaling and empirical absorption correction. The structure was solved by dual-space methods using the program SHELXT [74]. All non-hydrogen atoms were refined with anisotropic thermal parameters by full-matrix least-squares calculations on F^2^ using the program SHELXL-2014 [75]. Hydrogen atoms were inserted at calculated positions and constrained with isotropic thermal parameters except for the hydrogen atom of the hydroxyl group, which was located from a Fourier-difference map and refined isotopically. Drawings were produced with OLEX [76].

### 3.6. Computational Details

DFT calculations were performed with the Gaussian 16 program package (version B.01) [77]. The [Mn(***t*O2DO2A**)(H_2_O)] and [Mn(***t*O2DO2AM^Pip^**)(H_2_O)]^2+^ complexes were optimized using the hybrid-meta Generalized Gradient Approximation (GGA) functional M11 in combination with the def2-TZVP basis set [78,79]. Bulk water solvent effects were introduced using a polarized continuum model (integral equation formalism variant, IEFPCM) [80]. The stationary points were characterized using frequency calculations.

## 4. Conclusions

Two tDO2A-analogue complexing agents were synthesized in this work and their Mn(II) complexes were investigated. The ligand ***t*O2DO2A^2−^** can be viewed as a ***t*DO2A^2−^** chelator, where two unsubstituted (secondary) nitrogen atoms in the macrocycle are replaced for oxygen atoms, while ***t*O2DO2AM^Pip^** is its bis(piperidyl) amide. These structural changes allowed us to gather information on how the hardness of the donor atoms present in the macrocycle (nitrogen vs. oxygen atom) and the nature of the pendant arms (acetate vs. tertiary amide) affect the structure, the stability, the inertness and the relaxation properties of the Mn(II) complexes. The stability of the complexes of ***t*O2DO2A^2−^** with biogenic metal and Mn(II) ions match the literature data considering the different ligand basicity at different ionic strength (this was also confirmed by the pMn values of the Mn(II) complexes). The stability constants of the complexes formed with ***t*O2DO2AM^Pip^** are lower than those with ***t*O2DO2A^2−^**, following the different basicity of the ligands, which is a consequence of the replacement of negatively charged acetate groups by neutral amide pendants. Cu(II) and Mn(II) complexes formed with the ***t*O2DO2A^2−^** ligand were grown as single crystals and their structures determined by X-ray crystallography. The coordination polyhedron in Cu(II) complex can be described as distorted octahedral, while capped trigonal prismatic coordination fashion can be suggested for the Mn(II) chelate, in which seventh coordination site is occupied by a coordinated water oxygen. Water coordination in the [Mn(***t*O2DO2A**)] complex is also supported by relaxivity measurements. Water exchange on [Mn(***t*O2DO2A**)] is relatively slow and gets even slower upon the replacement of the two acetate pendants by tertiary amides in ***t*O2DO2AM^Pip^**. The inertness of the [Mn(***t*O2DO2A**)] and [Mn(***t*O2DO2AM^Pip^**)] complexes were probed by studying metal exchange reactions occurring with biogenic Cu(II) ions. The presence of a coordinated water molecule in [Mn(***t*O2DO2A**)] does not affect its kinetic inertness much, as the complex dissociates with similar rate as [Mn(***t*DO2A**)]. As expected, the replacement of two negatively charged acetate pendants by neutral amide moieties in ***t*O2DO2AM^Pip^** slows down the acid assisted dissociation of its Mn(II) complex. However, the spontaneous dissociation gets pronounced for the amide derivative, resulting in rapid dissociation of the complex at near neutral pH. Altogether, the 1,7-diaza-12-crown-4 macrocycle may be a potential candidate for further ligand development, but the inertness of the Mn(II) complex needs to be improved. In contrast to the literature data on other types of amide derivative ligands, here the substitution of carboxylates with amide functions in the ligand is not favorable for the kinetic inertness of the Mn(II) complex.

## Figures and Tables

**Table 1:** Bond distances of the metal coordination environments in [Mn(***t*O2DO2A**)(H_2_O)] and [Cu(***t*O2DO2A**)] complexes obtained from X-ray diffraction studies.

Mn(1)–O(1)	2.3124(17)	Cu(1)–O(1)	2.3783(8)
Mn(1)–O(2)	2.4542(17)	Cu(1)–O(2)	2.3282(8)
Mn(1)–O(3)	2.1518(16)	Cu(1)–O(3)	1.9426(8)
Mn(1)–O(5)	2.1566(17)	Cu(1)–O(5)	1.9356(8)
Mn(1)–N(1)	2.3756(19)	Cu(1)–N(1)	2.1036(9)
Mn(1)–N(2)	2.3855(18)	Cu(1)–N(2)	2.0763(9)
Mn(1)–O(1W)	2.2054(18)		-

**Table 2:** Protonation constants of ***t*O2DO2A^2−^** and ***t*O2DO2AM^Pip^** (*I* = 0.15 M NaCl, T = 25 °C) and related ligands provided for comparison.

	*t*O2DO2A^2−^	*t*O2DO2AM^Pip^	*t*DO2A^2−^	*t*PC2A^2−^
log*K*_1_^H^	8.05(2)/9.53 *^a^*	7.19(2)	11.69 *^b^*/11.29 *^c^*/11.66 *^d^*	12.25 *^e^*/12.50 *^f^*
log*K*_2_^H^	7.43(2)/7.46 *^a^*	6.29(2)	9.75 *^b^*/9.84 *^c^*/9.75 *^d^*	5.97 *^e^*/5.75 *^f^*
log*K*_3_^H^	2.06(2)/2.11 *^a^*	-	3.97 *^b^*/3.97 *^c^*/4.06 *^d^*	3.47 *^e^*/3.28 *^f^*
log*K*_4_^H^	-	-	2.68 *^b^*/2.59 *^c^*/2.58 *^d^*	1.99 *^e^*/2.38 *^f^*
∑log*Ki*^H^	17.54/19.10 *^a^*	13.48	28.09 *^b^*/27.69 *^c^*/28.05 *^d^*	23.68 *^e^*/23.91 *^f^*
∑log*K*_i_^H^, *i* = 1–2	15.48/16.99 *^a^*	13.48	21.44 *^b^*/21.13 *^c^*/21.41 *^d^*	18.22 *^e^*/18.25 *^f^*

*^a^* Data in 0.1 M Me_4_NNO_3_ from ref. [49]. *^b^* Data in 0.15 M NaCl from ref. [17]. *^c^* Data in 0.1 M Me_4_NCl from Ref. [18]. *^d^* Data in 0.1 M KCl from Ref. [17]. *^e^* Data in 0.15 M NaCl from Ref. [19]. *^f^* Data in 0.1 M NaCl from Ref. [48].

**Table 3:** Stability and protonation constants of the complexes formed with ***t*O2DO2A^2−^**, ***t*O2DO2AM^Pip^** (*I* = 0.15 M NaCl, T = 25 °C) and related ligands provided for comparison.

	*t*O2DO2A^2−^	*t*O2DO2AM^Pip^	*t*DO2A^2−^	*t*PC2A^2−^
log*K*_MgL_	3.91(1)/5.62 ^*a*^	2.91(5)	-	9.84 *^e^*
log*K*_CaL_	6.96(1)/8.50 ^*a*^	5.78(1)	8.86 *^d^*	9.82 *^e^*
log*K*_MnL_	9.38(1)/11.03 ^*a*^	7.76(3)	14.64 *^b^*/14.54 ^*c*^/15.07 *^d^*	17.09*^e^*
log*K*_MnHL_	-	-	4.40 *^b^*/4.25 *^c^*/4.48 *^d^*	2.14 *^e^*
log*K*_MnH2L_	-	-	4.45 *^c^*	-
log*K*_MnLOH_	12.38(2)	-	11.50 *^c^*	-
log*K*_ZnL_	10.55(3)/12.28 ^*a*^	8.69(2)	18.86 *^d^*	19.49 *^e^*
log*K*_ZnHL_	-	-	4.23 *^d^*	2.74 *^e^*
log*K*_ZnH2L_	-	-	1.78 *^d^*	-
log*K*_ZnLOH_	11.40(4)	-	-	-
log*K*_CuL_	14.56(2)/15.95 ^*a*^	10.66(3)	24.24 *^d^*	23.58 *^e^*
log*K*_CuHL_	2.46(3)	-	3.06 *^d^*	2.12 *^e^*
log*K*_CuLOH_	11.95(3)	-	-	-
pMn *^e^*	6.67/6.79 ^*a*^	6.28	6.52 *^b^*/6.60 *^c^*/6.73 *^d^*	8.64 *^e^*

*^a^* Data in 0.1 M Me_4_NNO_3_ from Ref. [49]. *^b^* Data in 0.15 M NaCl from ref. [17]. *^c^* Data in 0.1 M Me_4_NCl ref. [18]. *^d^* Data in 0.1 M KCl from refs. [17,27]]. *^e^* Defined as –log[Mn^2+^]_free_ for *c*_L_ = *c*_Mn_ = 10^−5^ M, pH 7.4.

**Table 4:** Rate and equilibrium constants and half-lives of dissociation (pH = 7.4) determined for the Mn(II) complexes of ***t*O2DO2A^2^**^−^ and ***t*O2DO2AM^Pip^** (T = 25 °C and *I* = 0.15 M NaCl) and related systems provided for comparison.

	*t*O2DO2A^2−^	*t*O2DO2AM^Pip^	*t*DO2A^2−^*^a^*	*t*PC2A^2−^*^b^*
*k*0 (s^−1^)	-	(1.54 ± 0.01) × 10^−3^	-	-
*k*1 (M^−1^s^−1^)	100 ± 5	2.3 ± 0.06	85	221
*k*2 (M^−2^s^−1^)	-	-	3 × 10^6^	-
*k*3 (M^−1^s^−1^)	(9 ± 1) × 10^−2^	-	-	3.6 × 10^−2^
log*K*_Mn(HL)_	3.4 ± 0.1	-	4.4	3.6
log*K*_M(L)M_	1.2 ± 0.2	-	-	1.4
*t*_1/2_ (h) *^c^*	39	0.13	58	21

*^a^* Data in 0.1 M KCl from Ref. [18]. *^b^* Data in 0.15 M NaCl from ref. [19]. *^c^* Half-lives of dissociation calculated at pH = 7.4 by using the rate constants and 0.01 mM Cu(II) ion concentration.

**Table 5:** Best-fit parameters obtained from the analysis of reduced relaxation ^17^O NMR rates and NMRD profiles of [Mn(***t*O2DO2A**)(H_2_O)] and [Mn(***t*O2DO2AM^Pip^**)(H_2_O)]^2+^ recorded at 9.4 T and related ligands.

	*t*O2DO2A^2−^	*t*O2DO2AM^Pip^	*t*PC2A^2−^^*e*^
*k*_ex_^298^/10^6^ s^−1^	53.3 ± 0.9	34.7 ± 0.3	126
Δ*H*^‡^/kJ mol^−1^ *^a^*	29.7 ± 0.6	28.8 ± 0.3	37.5
Δ*S*^‡^/J mol^−1^ K^−1^ *^a^*	2.8 ± 1.6	−3.9 ± 1.0	
*A*_O_/*ħ* / 10^6^ rad s^−1^ *^a^*	−45.3 ± 0.6	−40.1 *^c^*	−42.4
1/*T*_1_*e*^298^/10^8^ s^−1^ *^a^*	0.72 ± 0.40	0.18 ± 0.02	1.1
*τ*_R_^298^/ps *^b^*	46.7 ± 0.9	87 ± 1	
*E*_R_/kJ mol^−1^	20 *^d^*	19.3 ± 0.5	
Δ^2^/10^19^ rad s^−2^	11.1 ± 11	0.25 ± 0.1	

*^a^* From fits of the ^17^O NMR data. *^b^* From fits of the ^1^H NMRD data. *^c^* Fixed during the fit to the value obtained with DFT. *^d^* Fixed. *^e^* From Ref. [19].

**Table 6:** Crystal data and refinement details.

	[Cu(*t*O2DO2A)]·2H_2_O	[Mn(*t*O2DO2A)(H_2_O)]·2H_2_O
formula	C_12_H_24_CuN_2_O_8_	C_12_H_26_MnN_2_O_9_
MW	387.87	397.29
crystal system	monoclinic	orthorhombic
space group	P21/n	Pca21
T/K	100(2)	100(2)
a/Å	11.2189(5)	13.5220(3)
b/Å	11.8336(6)	16.7031(4)
c/Å	12.3596(6)	14.2920(3)
α/deg	90	90
β/deg	112.853(2)	90
γ/deg	90	90
V/Å^3^	1512.06(13)	3227.98(13)
F(000)	812	1.672
Z	4	8
λ, Å (Mo Kα)	0.71073	0.71073
D_calc_/g·cm^−3^	1.704	1.635
μ/mm^−1^	1.490	0.869
*θ* range/deg	2.48–30.51	2.85–30.32
R_int_	0.0279	0.0490
reflns obsd	4618	9852
GOF on *F*^2^	1.063	1.033
R1 ^a^	0.0204	0.0304
*w*R2 (all data) ^b^	0.0544	0.0636
Largest differences peax and hole/e Å^−3^	0.472 and −0.352	0.359 and −0.365

^a^ R_1_ = ∑||F_o_|-|F_c_||/∑|F_o_|. ^b^ wR_2_ (all data) = {∑[w(||F_o_|^2^-|F_c_|^2^)^2^]/∑[w(F_o_^4^)]}^1/2^.

## Data Availability

All Supplementary Materials can be found at MDPI. Our data will also be made available to any investigator upon request.

## Supplementary Materials

The following are available online: NMR and ESI-MS data of the synthesized compounds, details on the dissociation kinetics experiments, equations used for the fitting of ^17^O NMR and ^1^H NMRD data, details of the DFT calculated structures. CCDC 2044167 and 2044168 contain the supplementary crystallographic data for this paper. These data can be obtained free of charge via http://www.ccdc.cam.ac.uk/conts/retrieving.html (or from the CCDC, 12 Union Road, Cambridge CB2 1EZ, UK; Fax: +44 1223 336033; E-mail: deposit@ccdc.cam.ac.uk). For Supplementary Materials and crystallographic data in CIF or other electronic format see DOI.

## Sample Availability

Samples of the compounds are not available available from the authors.
