# Supplementary material for: Expanding the Ligand Classes Used for Mn(II) Complexation: Oxa-aza Macrocycles Make the Difference
Source: Molecules. 2021 Mar 10;26(6):1524. doi: 10.3390/molecules26061524 (PMC7998310; doi:10.3390/molecules26061524)
Supplement: Supplementary file 1 [file molecules-26-01524-s001.pdf]

Supplementary Materials for

Expanding the ligand classes used for Mn(II) complexation: Oxa-aza macrocycles make the difference

Ferenc K. Kálmán [1], Viktória Nagy [1], Rocío Uzal-Varela [2], Paulo Pérez-Lourido [3], David Esteban-Gómez [2], Zoltán Garda [1], Kristof Pota [4], Roland Mezei [1], Agnès Pallier,[5] Éva Tóth [5,*], Carlos Platas-Iglesias [2,*] and Gyula Tircsó [1,*]

[1] Department of Physical Chemistry, University of Debrecen, H-4010, Debrecen, Egyetem tér 1., Hungary. kalman.ferenc@science.unideb.hu (F.K.K.); nagywiki@gmail.com (V.N.); garda.zoltan@science.unideb.hu (Z.G.); mailod@freemail.hu (R.M.) gyula.tircso@science.unideb.hu (G.T.)

[2] Universidade da Coruña, Centro de Investigacións Científicas Avanzadas (CICA) and Departamento de Química, Facultade de Ciencias, 15071, A Coruña, Galicia, Spain. rocio.uzal@udc.es (R.U.-V.).; david.esteban@udc.es (D.E.-G.); carlos.platas.iglesias@udc.es (C.P.-I.)

[3] Departamento de Química Inorgánica, Facultad de Ciencias, Universidade de Vigo, As Lagoas, Marcosende, 36310 Ponte-vedra, Spain. paulo@uvigo.es

[4] Department of Inorganic and Analytical Chemistry, University of Debrecen, H-4010, Debrecen, Egyetem tér 1., Hungary. Current address of the author: Texas Christian University, Department of Chemistry and Biochemistry, 2950 West Bowie Street, Fort Worth, Texas 76109, USA, kristof.pota@tcu.edu

[5] Centre de Biophysique Moléculaire, CNRS, Rue Charles-Sadron, 45071 Orleans Cedex 2, France. agnes.pallier@cnrs.fr (A.P.); eva.jakabtoth@cnrs.fr (E.T.)

* Correspondence: carlos.platas.iglesias@udc.es (C.P.-I.); eva.jakabtoth@cnrs.fr (E.T.) and gyula.tircso@science.unideb.hu (Gy.T.)

Summary

**Scheme S1**	Synthesis of the ligands investigated in this work. Reagents and conditions: i) CH_3CN, 65 ºC, K_2CO_3, KI; ii) CH_3CN, 45 ºC, Na_2CO_3, KI; iii) TFA: CH_2Cl_2, reflux.	
**Figure S1.**	^{1}H (500 MHz, 25 °C, top) and ^{13}C (125.8 MHz, 25 °C, bottom) NMR spectra of compound **1** recorded in $CDCl_3$ solution.	3
**Figure S2.**	^{1}H (500 MHz, 25 °C, pD 7.0, top) and ^{13}C (125.8 MHz, 25 °C, pD 7.0, bottom) NMR spectra of H_2***t*****O2DO2A**·$2CF_3COOH·H_2O$ recorded in D_2O solution.	4
**Figure S3.**	^{1}H NMR spectrum (top) and {^{1}H}^{13}C NMR spectrum of the ***t*****O2DO2AM^{pip}** recorded in D_2O using Bruker DRX 360 NMR spectrometer at 25 ^{o}C.	5
**Figure S4.**	ESI-MS spectra of the ***t*****O2DO2AM^{pip}** chelator.	6
**Figure S5.**	Coordination polyhedra around the metal ions in the X-ray crystal structures of [Mn(***t*****O2DO2A**)(H_2O)] (left) and [Cu(***t*****O2DO2A**)] (right).	7
**Figure S6.**	Views of the structures of the [Mn(***t*****O2DO2A**)(H_2O)] (left) and [Mn(***t*****O2DO2AM^{pip}**)(H_2O)]$^{2+}$ (right) complexes obtained with DFT calculations at the M11/def2-TZVP level.	7
**Table S1.**	Bond distances (Å) of the metal coordination environments in [Mn(***t*****O2DO2A**)(H_2O)] and [Mn(***t*****O2DO2AM^{pip}**)(H_2O)]$^{2+}$ complexes obtained with DFT calculations at the M11/def2-TZVP level.	8
**Table S2.**	Optimized Cartesian coordinates (Å) of [Mn(***t*****O2DO2A**)(H_2O)] obtained with DFT calculations at the M11/def2-TZVP level.	8
**Table S3.**	Optimized Cartesian coordinates (Å) of [Mn(***t*****O2DO2AM^{pip}**)(H_2O)]$^{2+}$ obtained with DFT calculations at the M11/def2-TZVP level.	9
	Dissociation kinetics of the Mn(II) complexes	11
	Measurements of ^{17}O NMR relaxation rates	12
	References	14

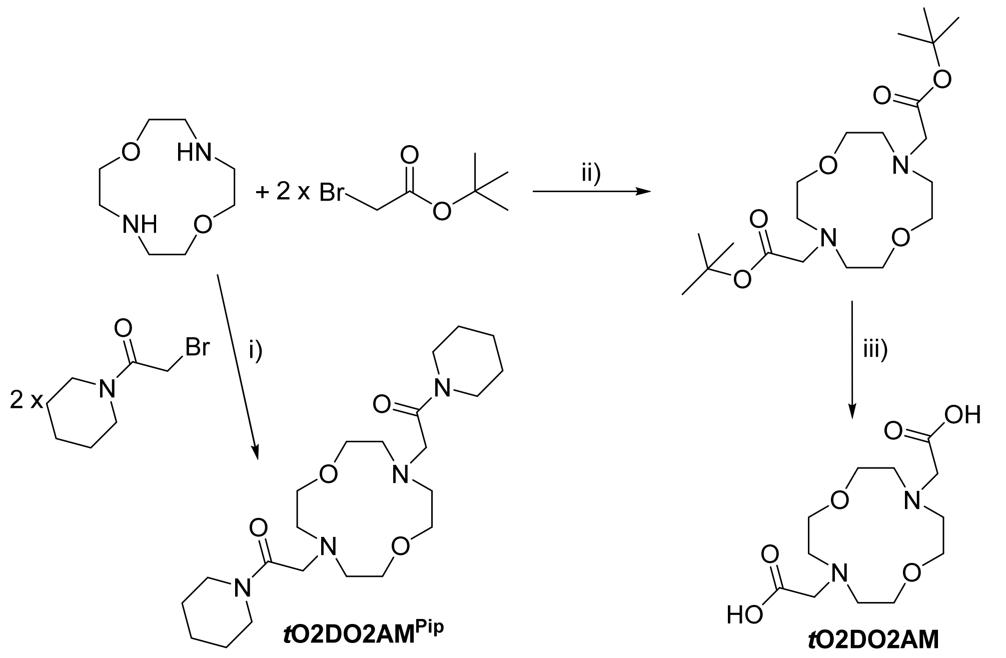

Scheme S1. Synthesis of the ligands investigated in this work. Reagents and conditions: i) CH_3CN, 65 ºC, K_2CO_3, KI; ii) CH_3CN, 45 ºC, Na_2CO_3, KI; iii) TFA: CH_2Cl_2, reflux.

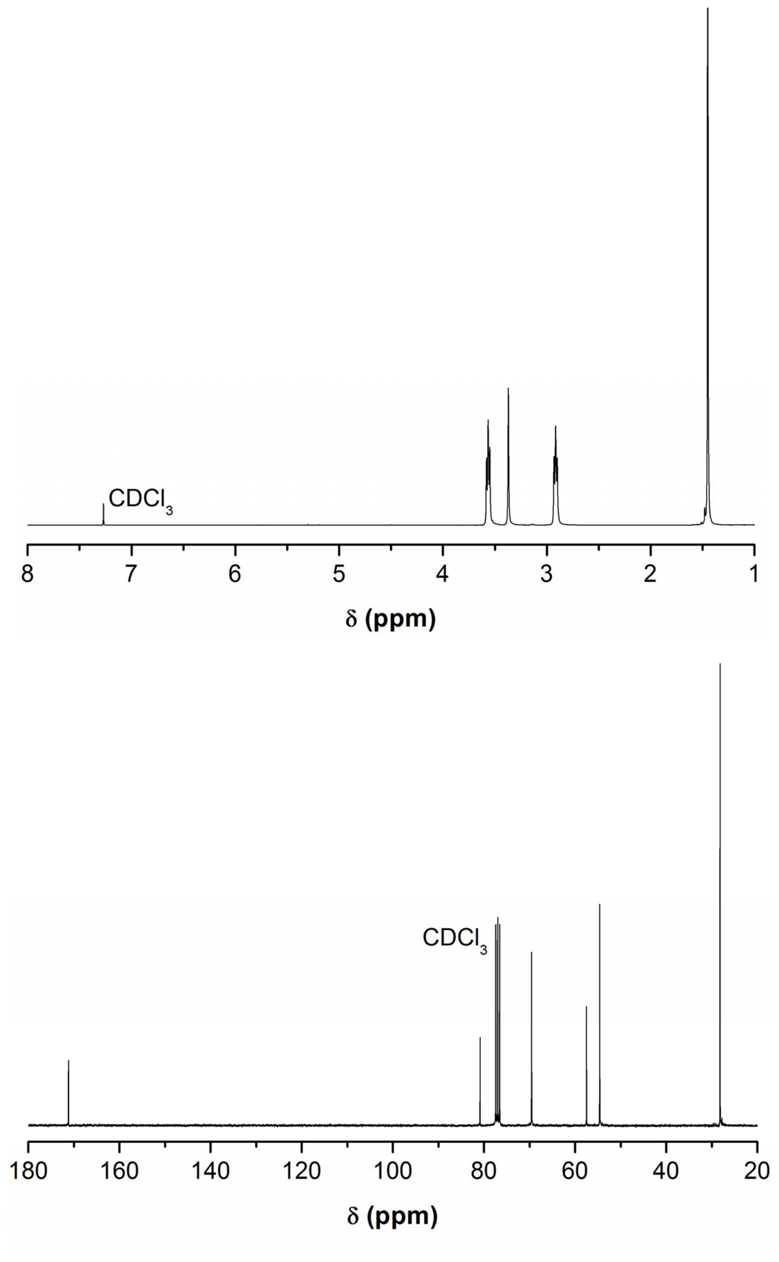

Figure S1. ^{1}H (500 MHz, 25 °C, top) and ^{13}C (125.8 MHz, 25 °C, bottom) NMR spectra of compound **1** recorded in $CDCl_3$ solution.

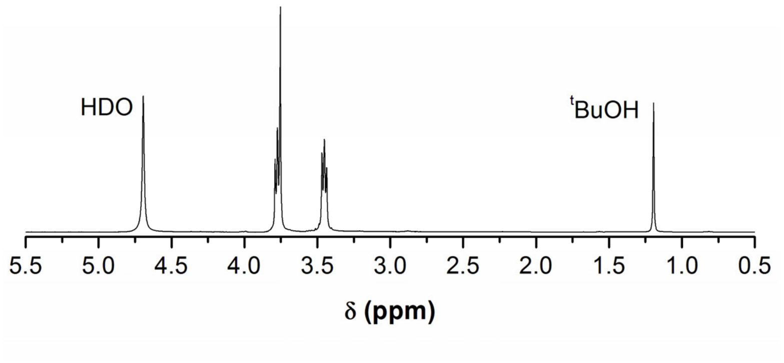

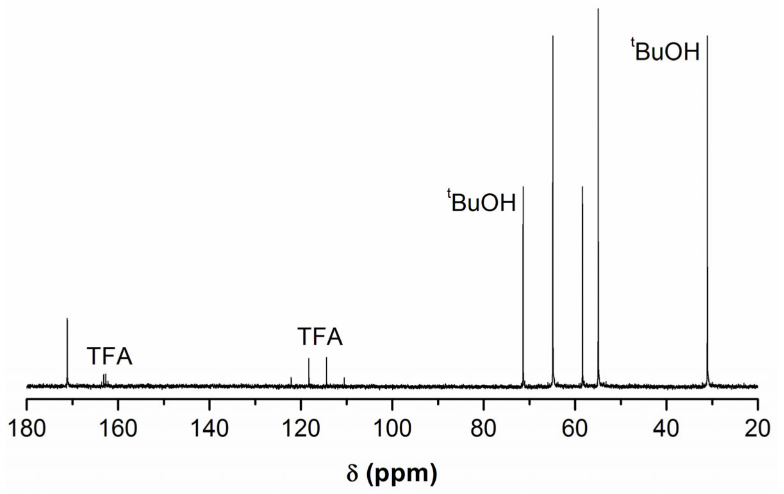

Figure S2. ^{1}H (500 MHz, 25 °C, pD 7.0, top) and ^{13}C (125.8 MHz, 25 °C, pD 7.0, bottom) NMR spectra of H_2***tO2DO2A***·$2CF_3COOH$·H_2O recorded in D_2O solution.

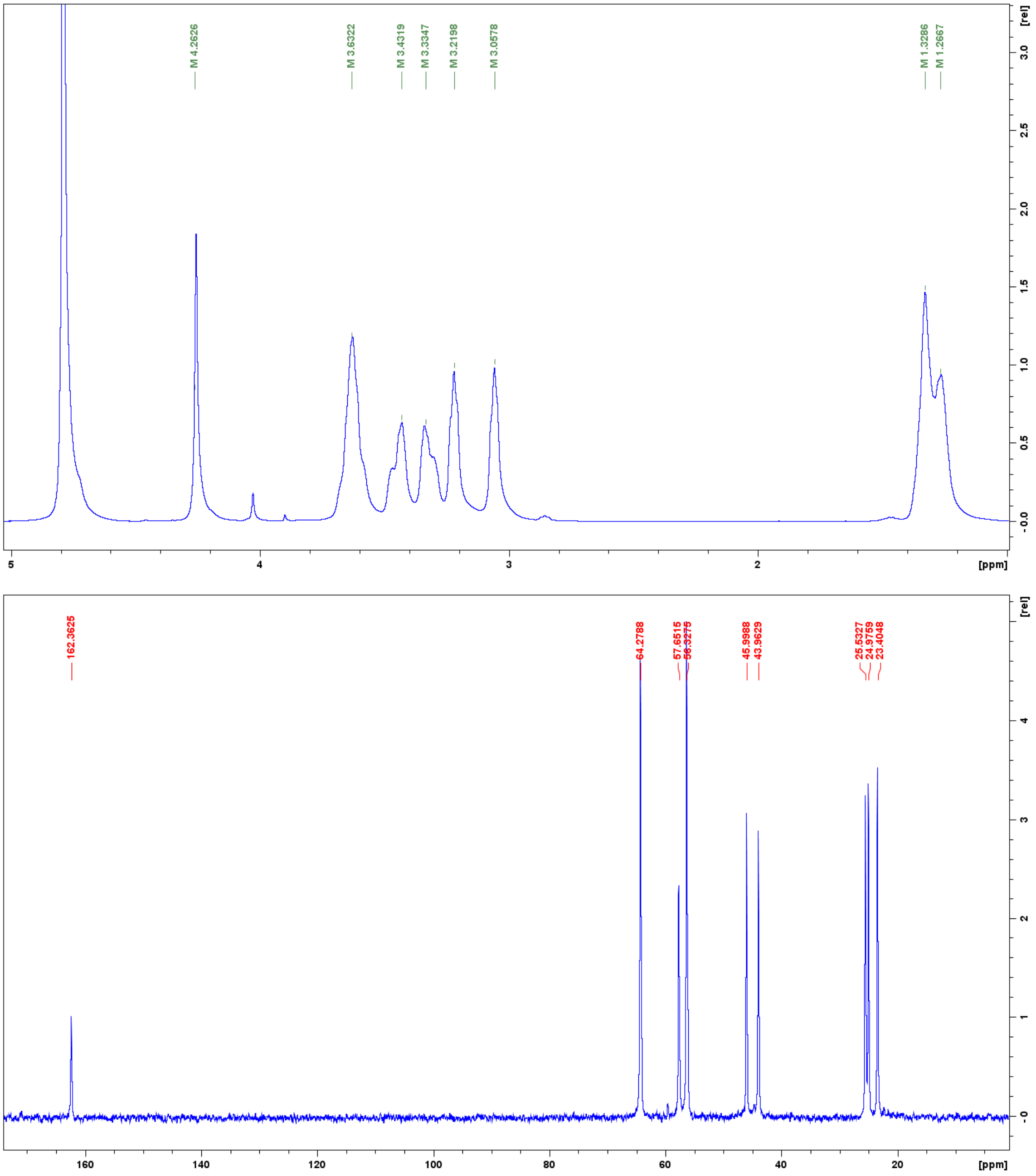

Figure S3. ^{1}H NMR spectrum (top) and {^{1}H}^{13}C NMR spectrum of the ***t*O2DO2AM^{Pip}** recorded in D_2O using Bruker DRX 360 NMR spectrometer at 25 ^{o}C.

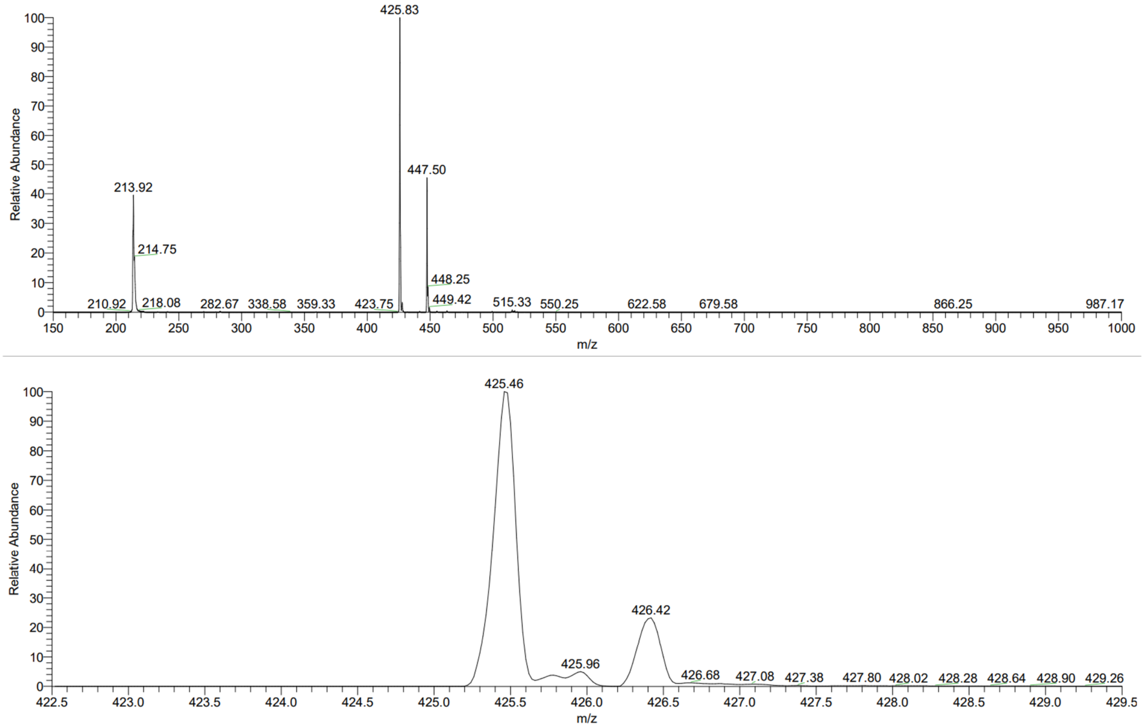

Figure S4. ESI-MS spectra of the ***t*O2DO2AM**$^{\mathbf{Pip}}$ chelator.

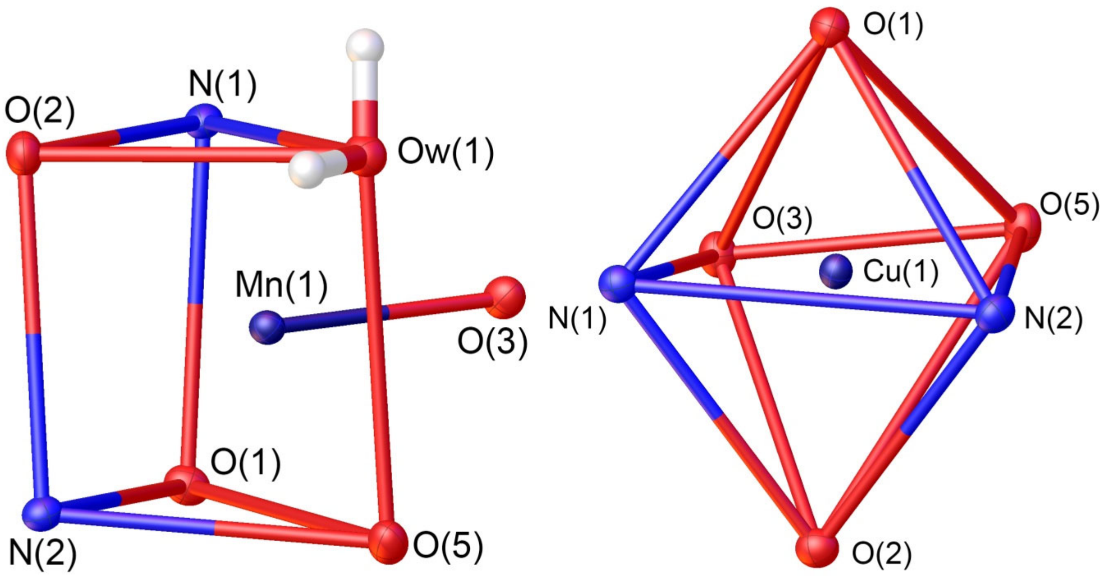

Figure S5. Coordination polyhedra around the metal ions in the X-ray crystal structures of [Mn(***t*O2DO2A**)(H_2O)] (left) and [Cu(***t*O2DO2A**)] (right).

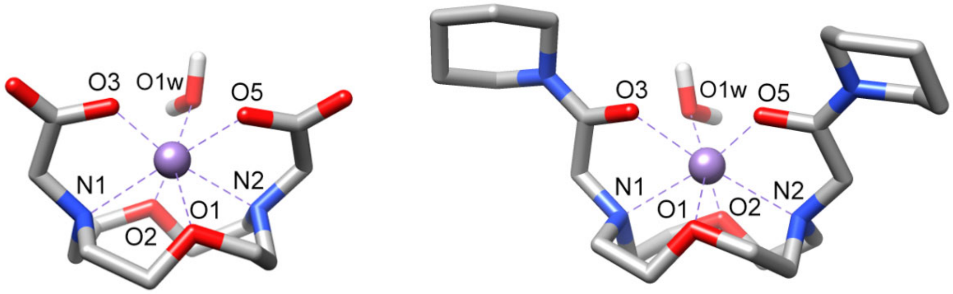

Figure S6. Views of the structures of the [Mn(***t*O2DO2A**)(H_2O)] (left) and [Mn(***t*O2DO2AM**$^{\mathbf{pip}}$)(H_2O)]$^{2+}$ (right) complexes obtained with DFT calculations at the M11/def2-TZVP level.

Table S1. Bond distances (Å) of the metal coordination environments in [Mn(***t*O2DO2A**)(H_2O)] and [Mn(***t*O2DO2AM**^{pip})(H_2O)]$^{2+}$ complexes obtained with DFT calculations at the M11/def2-TZVP level.

	**[Mn(tO2DO2A)]**	**[Mn(tO2DO2Am^{pip})]$^{2+}$**
Mn1-N1	2.420	2.379
Mn1-N2	2.425	2.444
Mn1-O1	2.339	2.323
Mn1-O2	2.441	2.354
Mn1-O3	2.151	2.233
Mn1-O5	2.162	2.159
Mn1-O1w	2.281	2.293

Table S2. Optimized Cartesian coordinates (Å) of [Mn(***t*O2DO2A**)(H_2O)] obtained with DFT calculations at the M11/def2-TZVP level.

Center Number	Atomic Number	Coordinates (Angstroms) X	Y	Z
1	25	0.036577	-0.460510	-0.381050
2	6	2.121001	0.241346	1.886360
3	1	2.931937	0.800965	2.375923
4	1	2.316796	-0.827898	2.025095
5	6	0.796032	0.560531	2.543965
6	1	0.847484	0.353551	3.620009
7	1	0.517740	1.615898	2.411607
8	6	-1.534299	0.009709	2.310405
9	1	-1.572631	0.309308	3.364518
10	1	-2.095266	-0.920648	2.194322
11	6	-2.092537	1.116426	1.429780
12	1	-3.132281	1.330760	1.721524
13	1	-1.516652	2.030120	1.605204
14	6	-2.029184	1.989283	-0.835048
15	1	-2.819258	2.687228	-0.519797
16	1	-2.235229	1.684858	-1.865521
17	6	-0.690046	2.695644	-0.818406
18	1	-0.724357	3.581714	-1.464602
19	1	-0.408385	3.022824	0.192162
20	6	1.630795	2.244558	-1.236704
21	1	1.669660	3.320706	-1.443574
22	1	2.181548	1.725331	-2.026729
23	6	2.212634	1.941228	0.135517
24	1	3.260095	2.275750	0.181630
25	1	1.662094	2.506030	0.894166
26	6	3.149076	-0.269610	-0.222021
27	1	4.102550	-0.200992	0.319696
28	1	3.316462	0.124268	-1.230485
29	6	2.769566	-1.750109	-0.394821
30	6	-3.072016	-0.163345	-0.383529
31	1	-3.231535	-0.086290	-1.464135
32	1	-4.023414	0.071195	0.112857
33	6	-2.734105	-1.644287	-0.136249
34	7	2.097646	0.507862	0.438014
35	7	-2.013525	0.776723	-0.000085

36	8	-0.693964	-0.492419	-2.541847
37	1	-0.813987	-1.292247	-3.063254
38	8	-0.184560	-0.282031	1.940686
39	8	0.283154	1.779544	-1.319375
40	8	1.524921	-2.012700	-0.426982
41	8	3.686294	-2.563823	-0.522540
42	8	-1.503240	-1.951174	-0.092918
43	8	-3.682634	-2.429454	-0.042259
44	1	-0.337921	0.196438	-3.113494

E(UM11) = -2257.6801136 Hartree
Zero-point correction = 0.361880
Thermal correction to Energy = 0.384850
Thermal correction to Enthalpy = 0.385794
Thermal correction to Gibbs Free Energy = 0.309794
Sum of electronic and zero-point Energies = -2257.318234
Sum of electronic and thermal Energies = -2257.295264
Sum of electronic and thermal Enthalpies = -2257.294319
Sum of electronic and thermal Free Energies = -2257.370320

Table S3. Optimized Cartesian coordinates (Å) of [Mn(***t*****O2DO2AM**$^{\mathbf{pip}}$)(H_2O)]$^{2+}$ obtained with DFT calculations at the M11/def2-TZVP level.

Center Number	Atomic Number	Coordinates (Angstroms) X	Y	Z
1	25	0.009261	-0.697518	-0.360499
2	6	-1.854757	-1.316901	2.090674
3	1	-2.597022	-1.899809	2.654709
4	1	-2.089151	-0.255410	2.215964
5	6	-0.471860	-1.559396	2.654654
6	1	-0.446033	-1.276532	3.713608
7	1	-0.176231	-2.614297	2.576694
8	6	1.821575	-1.010918	2.188635
9	1	1.950716	-1.269797	3.245590
10	1	2.366580	-0.081812	1.994945
11	6	2.305439	-2.145379	1.302372
12	1	3.347417	-2.398393	1.547696
13	1	1.702936	-3.034219	1.508230
14	6	2.162489	-3.022098	-0.970362
15	1	2.999784	-3.690343	-0.722919
16	1	2.272574	-2.706046	-2.011824
17	6	0.858290	-3.784984	-0.854164
18	1	0.865403	-4.627708	-1.555709
19	1	0.692142	-4.185939	0.154442
20	6	-1.519021	-3.404663	-1.005598
21	1	-1.523752	-4.486755	-1.175207
22	1	-2.149468	-2.934962	-1.766385
23	6	-2.004782	-3.079548	0.397500
24	1	-3.025788	-3.460575	0.543448
25	1	-1.363290	-3.585812	1.125682
26	6	-3.064104	-0.920263	0.054004
27	1	-3.954115	-1.012646	0.691316
28	1	-3.303024	-1.350198	-0.924530
29	6	-2.703998	0.550888	-0.159686
30	6	3.221410	-0.870629	-0.542097
31	1	3.535134	-1.092625	-1.567465
32	1	4.107676	-0.980178	0.095324

33	6	2.748202	0.582667	-0.520902
34	7	-1.932346	-1.630148	0.648469
35	7	2.173562	-1.808414	-0.128143
36	8	-1.083174	-0.626663	-2.375262
37	1	-0.765667	-1.246687	-3.041901
38	8	0.442379	-0.740624	1.920868
39	8	-0.197588	-2.886957	-1.199535
40	8	-1.512891	0.903554	-0.034343
41	8	1.533890	0.830604	-0.367928
42	1	-1.271243	0.209148	-2.816301
43	7	-3.664351	1.399937	-0.485076
44	7	3.641727	1.542918	-0.679438
45	6	-5.074498	1.052176	-0.670930
46	1	-5.350091	1.358657	-1.688685
47	1	-5.215090	-0.025582	-0.605698
48	6	-3.386426	2.829761	-0.645813
49	1	-2.315499	2.985852	-0.525124
50	1	-3.673512	3.110783	-1.667670
51	6	5.081548	1.342689	-0.860754
52	1	5.361701	1.852757	-1.791550
53	1	5.310984	0.286092	-0.987318
54	6	3.254796	2.954991	-0.600358
55	1	3.557983	3.434357	-1.540105
56	1	2.171178	3.009758	-0.510186
57	6	5.839056	1.953264	0.315817
58	6	3.965840	3.610802	0.581092
59	1	3.604791	3.150267	1.509642
60	1	3.699518	4.671678	0.613268
61	6	-4.203536	3.626809	0.368356
62	1	-3.857416	3.373588	1.378399
63	1	-4.018926	4.694306	0.213890
64	6	-5.931921	1.800487	0.346349
65	1	-5.671660	1.453323	1.354446
66	1	-6.984669	1.557762	0.172866
67	6	-5.692498	3.306428	0.233092
68	1	-6.268992	3.842085	0.993259
69	1	-6.047815	3.652621	-0.747231
70	6	5.480097	3.432108	0.465877
71	1	5.982370	3.858907	1.339184
72	1	5.841186	3.979598	-0.415615
73	1	6.913553	1.826259	0.152950
74	1	5.574187	1.407357	1.230698

E(UM11) = -2609.3972717 Hartree
Zero-point correction = 0.658061
Thermal correction to Energy = 0.690586
Thermal correction to Enthalpy = 0.691530
Thermal correction to Gibbs Free Energy = 0.593482
Sum of electronic and zero-point Energies = -2608.739211
Sum of electronic and thermal Energies = -2608.706686
Sum of electronic and thermal Enthalpies = -2608.705742
Sum of electronic and thermal Free Energies = -2608.803789

Dissociation Kinetics of the Mn(II) complexes

The metal-exchange reactions of the Mn(II) complexes can occur via associative or dissociative mechanisms. In the associative mechanisms, the Mn(II) chelate is attacked by the exchanging metal ion or its hydroxo complex when a dinuclear intermediate forms, [Mn(L)M] or [Mn(L)(OH)M]. In this case the functional groups of the ligand transfer from the Mn(II) ion to the exchanging metal ion (usually Cu(II) or Zn(II)) in a step-by-step manner. On the other hand, the decomplexation frequently occurs via spontaneous and proton-assisted pathways followed by the fast complexation between the free ligand and the exchanging metal ion as it is shown on Scheme S1.

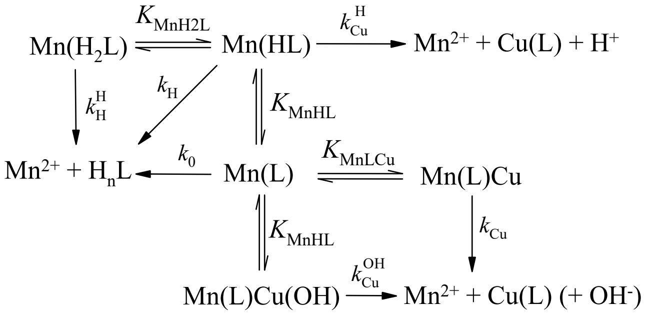

Scheme S1. Reaction mechanisms of the dissociation of Mn(II) complexes in metal exchange reactions (charges are omitted for clarity).

The k_0, k_H, k_H^H, and k_{Cu}, are the rate constants of the spontaneous, proton-assisted and metal-assisted reaction pathways, respectively. K_{MnHL}, K_{MnH_2L} and K_{MnLM} are the protonation constants of the complexes [MnL], [Mn(HL)] and the stability constant of the heterodinuclear complex [MnLCu], respectively.

Taking into account the possible pathways and the equations of K_{MnHL}, K_{MnH_2L} and K_{MnLM} the k_{obs} can be expressed by the following equation (presented in the manuscript):

$$k_{obs} = \frac{k_0 + k_1[\mathrm{H}^+] + k_2[\mathrm{H}^+]^2 + k_3[\mathrm{Cu}^{2+}]}{1 + K_{\mathrm{Mn(HL)}}[\mathrm{H}^+] + K_{\mathrm{Mn(HL)}}K_{\mathrm{Mn(H_2L)}}[\mathrm{H}^+]^2 + K_{\mathrm{Mn(L)Cu}}[\mathrm{Cu}^{2+}]} \quad \text{(S1)}$$

where K_{MnHL}=[Mn(HL)]/[MnL][H^+], K_{MnH_2L} =[Mn(H_2L)]/[Mn(HL)][H^+], K_{MnLCu}=[Mn(L)Cu]/[MnL][Cu^{2+}], k_1=$k_H \cdot K_{MnHL}$, k_2= $k_H^H \cdot K_{MnHL} \cdot K_{MnH_2L}$ and k_3=$k_M \cdot K_{MnLCu}$.

Measurements of ^{17}O NMR relaxation rates

The Swift and Connick theory was used to analyze the ^{17}O NMR data to evaluate the reduced transverse ^{17}O relaxation rates calculated from the relaxation rates $1/T_2$ and $1/T_{2A}$ measured for the paramagnetic solutions and the diamagnetic reference [1]:

$$\frac{1}{T_{2r}} = \frac{1}{P_m}\left[\frac{1}{T_2} - \frac{1}{T_{2A}}\right] = \frac{1}{\tau_m}\frac{T_{2m}^{-2} + \tau_m^{-1} T_{2m}{}^{-1} + \Delta\omega_m^2}{(\tau_m^{-1} + T_{2m}^{-1})^2 + \Delta\omega_m^2} \qquad \text{(S2)}$$

$\Delta\omega_m$ is governed by the hyperfine or scalar coupling constant, $A_O/\hbar$, where B, S and g_L are the magnetic field, the electron spin and the isotropic Landé g factor (Equation (S3)).

$$\Delta\omega_m = \frac{g_L \mu_B S(S+1) B}{3 k_B T}\frac{A_O}{\hbar} \qquad \text{(S3)}$$

The ^{17}O transverse relaxation rate is mainly determined by the scalar contribution ($1/T_{2sc}$).

$$\frac{1}{T_{2m}} \cong \frac{1}{T_{2sc}} = \frac{S(S+1)}{3}\left(\frac{A_O}{\hbar}\right)^2 \tau_s \qquad \frac{1}{\tau_s} = \frac{1}{\tau_m} + \frac{1}{T_1} \qquad \text{(S4)}$$

The exchange rate, k_{ex}, (or inverse binding time, τ_m) of the inner sphere water molecule is assumed to obey the Eyring equation (Equation (S5)) where $\Delta S^{\ddagger}$ and $\Delta H^{\ddagger}$ are the entropy and enthalpy of activation for the exchange, and $^{298}k_{ex}$ is the exchange rate at 298.15 K.

$$\frac{1}{\tau_m} = k_{ex} = \frac{k_B T}{h}\exp\left\{\frac{\Delta S^{\ddagger}}{R} - \frac{\Delta H^{\ddagger}}{RT}\right\} = \frac{k_{ex}^{298} T}{298.15}\exp\left\{\frac{\Delta H^{\ddagger}}{R}\left(\frac{1}{298.15} - \frac{1}{T}\right)\right\} \qquad \text{(S5)}$$

For the fit of the ^{17}O T_2 data, an exponential function of the temperature dependency of $1/T_{1e}$ was used:

$$\frac{1}{T_{1e}} = \frac{1}{T_{1e}^{298}}\exp\left\{\frac{E_v}{R}\left(\frac{1}{T} - \frac{1}{298.15}\right)\right\} \qquad \text{(S6)}$$

The ^{1}H relaxivity ($mM^{-1}s^{-1}$) of the Mn(II) complexes is determined by the inner- and outer-sphere contributions (Equation (S7)):

$$r_1 = r_{1is} + r_{1os} \qquad \text{(S7)}$$

The inner-sphere term is given by Equation (S8), where q is the number of inner-sphere water molecules.

$$r_{1is} = \frac{1}{1000} \times \frac{q}{55.55} \times \frac{1}{T_{1m}^{H} + \tau_m} \qquad \text{(S8)}$$

In the longitudinal relaxation rate of inner sphere water protons, $1/T_{1m}^{H}$, the dipolar contribution dominates (Equation (S9)):

$$\frac{1}{T_{1m}^{H}} \cong \frac{1}{T_1^{DD}} = \frac{2}{15}\left(\frac{\mu_0}{4\pi}\right)^2 \frac{\hbar^2 \gamma_S^2 \gamma_I^2}{r_{MnH}^6} S(S+1)\left[\frac{3\tau_{d1H}}{1+\omega_I^2\tau_{d1H}^2} + \frac{7\tau_{d2H}}{1+\omega_S^2\tau_{d2H}^2}\right] \qquad \text{(S9)}$$

Here r_{MnH} is the effective distance between the Mn^{2+} electron spin and the water protons, ω_I is the proton resonance frequency, τ_{diH} is given by Equation S10, where τ_{RH} is the rotational correlation time of the Mn^{2+}–H_{water} vector:

$$\frac{1}{\tau_{diH}} = \frac{1}{\tau_m} + \frac{1}{\tau_{RH}} + \frac{1}{T_{ie}} \qquad i = 1, 2; \qquad \text{(S10)}$$

$$\tau_{RH} = \tau_{RH}^{298} \exp\left\{\frac{E_R}{R}\left(\frac{1}{T} - \frac{1}{298.15}\right)\right\} \qquad \text{(S11)}$$

The electronic relaxation is mainly governed by modulation of the transient zero-field splitting, and for the electron spin relaxation rates, $1/T_{1e}$ and $1/T_{2e}$, McMachlan has developed Equations (S12)–(S14), which were used in the fit of the NMRD data [2]:

$$\left(\frac{1}{T_{1e}}\right) = \frac{32}{25}\Delta^2\left(\frac{\tau_v}{1+\omega_S^2\tau_v^2} + \frac{4\tau_v}{1+4\omega_S^2\tau_v^2}\right) \qquad \text{(S12)}$$

$$\left(\frac{1}{T_{2e}}\right) = \frac{32}{50}\Delta^2\left[3\tau_v + \frac{5\tau_v}{1+\omega_S^2\tau_v^2} + \frac{2\tau_v}{1+4\omega_S^2\tau_v^2}\right] \tag{S13}$$

$$\tau_v = \tau_v^{298}\exp\left\{\frac{E_v}{R}\left(\frac{1}{T} - \frac{1}{298.15}\right)\right\} \tag{S14}$$

where Δ^2 is the trace of the square of the transient zero-field-splitting (ZFS) tensor, τ_v is the correlation time for the modulation of the ZFS with the activation energy E_v, and ω_S is the Larmor frequency of the electron spin.

The outer-sphere contribution to the overall relaxivity is described by Equation (S15), where N_A is the Avogadro constant, and J_{os} is a spectral density function (Equation (S16)).

$$r_{1os} = \frac{32N_A\pi}{405}\left(\frac{\mu_0}{4\pi}\right)^2\frac{\hbar^2\gamma_S^2\gamma_I^2}{a_{MnH}D_{MnH}}S(S+1)\left[3J_{os}(\omega_I, T_{1e}) + 7J_{os}(\omega_S, T_{2e})\right] \tag{S15}$$

$$J_{os}(\omega, T_{je}) = \mathrm{Re}\left[\frac{1+\frac{1}{4}\left(i\omega\tau_{MnH} + \frac{\tau_{MnH}}{T_{je}}\right)^{1/2}}{1+\left(i\omega\tau_{MnH} + \frac{\tau_{MnH}}{T_{je}}\right)^{1/2} + \frac{4}{9}\left(i\omega\tau_{MnH} + \frac{\tau_{MnH}}{T_{je}}\right) + \frac{1}{9}\left(i\omega\tau_{MnH} + \frac{\tau_{MnH}}{T_{je}}\right)^{3/2}}\right]$$

$j = 1, 2$ (S16)

The diffusion coefficient for the diffusion of a water proton away from a Mn^{2+} complex, D_{MnH}, obeys the exponential temperature dependence described by Equation (S17), with activation energy E_{MnH}:

$$D_{MnH} = D_{MnH}^{298}\exp\left\{\frac{E_{MnH}}{R}\left(\frac{1}{298.15} - \frac{1}{T}\right)\right\} \tag{S17}$$

References

1. Swift, T. J.; Connick, R. E. NMR-Relaxation Mechanisms of O17 in Aqueous Solutions of Paramagnetic Cations and the Lifetime of Water Molecules in the First Coordination Sphere. *J. Chem. Phys.* **1962**, *37*, 307–320. DOI: 10.1063/1.1701321.
2. McLachlan, A. D. Line widths of electron resonance spectra in solution. *Proc. R. Soc. London, Ser. A.* **1964**, *280*, 271–288. DOI: 10.1098/rspa.1964.0145
